# Functional sweet tea in China: species, sweet components and anti-metabolic disease effects

**DOI:** 10.3389/fnut.2026.1742961

**Published:** 2026-06-24

**Authors:** Zi-Yu Yan, Xi-Fan Wei, Gui-Ming Deng, Wen-Bo Yang, Rui Gui, Wen-Xuan Wang, Feng-Hua Kang, Jing Li, Yi-Heng Liu, Kang-Ping Xu, Yi-Kun Wang

**Affiliations:** 1 Xiangya School of Pharmaceutical Sciences, Hunan Key Laboratory of Diagnostic and Therapeutic Drug Research for Chronic Diseases, Central South University, Changsha, Hunan, China; 2 Hunan University of Chinese Medicine, The First Hospital of Hunan University of Chinese Medicine, Changsha, Hunan, China; 3 Department of Pharmacy, Xiangya Hospital of Central South University, Changsha, Hunan, China; 4 Orthopedic Center, Affiliated Haikou Hospital of Central South University Xiangya School of Medicine, Haikou, Hainan, China

**Keywords:** chronic metabolic disease, geographical distribution, species, sweet components, sweet tea

## Abstract

Functional sweet tea refers to tea beverages with health benefits and sweetness made from natural sweet plants, which has shown a promising potential for the prevention and treatment of various chronic metabolic diseases. However, no comprehensive catalogs of sweet tea have been reported, and the systematic synthesis of their species, distribution, life forms, and health benefits is lacking. In this review, we firstly define scientific concept functional sweet tea, referred to tea beverages with health benefits and sweetness made from natural sweet plants. A total of 22 functional sweet tea plants in China and 27 reported functional sweet components is firstly compiled, which not only standardizes academic terminology but also directly facilitates market growth of the sweet tea industry. Sweet components show strong potential on preventing hyperglycaemia, hyperlipidaemia, hypertension, hyperuricaemia, liver disease through facilitation of glycolipid metabolism, anti-inflammatory and antioxidant pathways, targeting multiple key mediators such as AMPK, SGLT, Nrf2, and intestinal flora. This review provides new insights into the prevention and management of various metabolic diseases through sweet tea, and holds important theoretical and practical significance for the development of functional foods, the industrialization of natural sweeteners, and the optimization of prevention and management strategies for metabolic diseases.

## Graphical abstract

**Figure:**
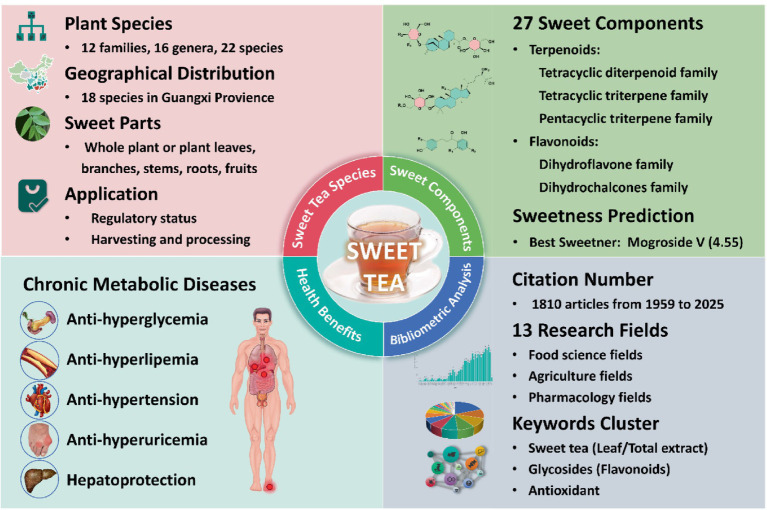

## 1 Introduction

Sweet tea, a naturally sweet-tasting plant integrating the attributes of tea, sweetener, and therapeutic agent, has garnered considerable attention within food science and natural medicinal chemistry over recent years. Sweet tea not only provides natural sweetness but also contains diverse bioactive constituents, exhibiting distinctive therapeutic potential for the prevention and management of metabolic diseases (1). Given the escalating global burden of obesity, diabetes, and cardiovascular diseases attributable to excessive sugar consumption, research on natural sweeteners has emerged as a priority area within food and health sciences (2). Despite substantial researches and commercial investments, diverse plant species and production origins affect distinct phytochemical profiles, quality and therapeutic properties of sweet tea. For instance, the predominant sweet tea species in Guangxi is *Rubus chingii* var. *suavissimus* (S. Lee) L. T. Lu leaves, whose primary sweet component is rubusoside, a tetracyclic diterpenoid glycoside (3). In Guangdong and Hunan, the commonly consumed sweet tea derives from *Lithocarpus litseifolius* (Hance) Chun leaves, containing dihydrochalcone derivatives including phlorizin and trilobatin as principal sweet components (4). Currently, the related directory of sweet tea species and resources has not been reported, and there is a lack of systematic review of the botanical characteristics, distribution, life form and health benefits of sweet tea, which limits the systematic development of sweet tea resources to a certain extent. Furthermore, sweet components in sweet tea modulate taste perception via intricate signaling networks involving T1R and T2R receptors and neurochemical pathways, yet the mechanistic basis of these interactions remains incompletely characterized.

Recent studies have demonstrated associations between erythritol/xylitol and elevated cardiovascular risk (1). Growing consumer skepticism toward artificial sweeteners (e.g., aspartame, sucralose) has accelerated demand for natural sweetener alternatives. Compared with conventional tea beverages, sweet tea serves simultaneously as both a “zero-calorie sugar substitute” and an “active pharmaceutical ingredient”. It not only enhances sweetness but also provides a higher nutrient content than that found in tea processed from *Camellia sinensis* L. In contrast to chemical synthetic sweeteners, sweet tea represents a natural, safe, low-calorie alternative with health benefits in metabolic disease prevention and management, which makes it serve both as a widely consumed functional beverage in traditional medicine systems and as an ideal raw material for sugar substitutes and nutraceuticals. Consequently, it serves as both a widely consumed functional beverage in traditional medicine systems and an ideal raw material for sugar substitutes and nutraceutical products. For instance, steviol glycosides and mogrosides have received Generally Recognized as Safe status from the U.S. FDA as natural sweeteners (5). The pathogenesis of metabolic diseases is inherently complex. Sweet tea demonstrates multi-target, multi-pathway regulatory characteristics in metabolic modulation, thereby transcending the limitations of conventional single-target therapeutics and offering novel strategies for comprehensive metabolic disease intervention. Nevertheless, current methodologies for investigating the molecular mechanisms of sweet tea remain relatively constrained, limiting both the depth and scope of research. Furthermore, molecular mechanism studies of sweet tea bioactive constituents have predominantly been conducted at cellular and animal levels. Only a limited number of sweet tea species (such as *L. litseifolius*, *G. uralensis*, and *S. rebaudiana*) have undergone partial mechanistic validation in humans, whereas the majority remain at the preclinical stage, presenting challenges for translational applicability to human physiology.

In this review, a systematic synthesis of sweet tea types, resources, geographical distribution, and sweet components has been provided. We have analyzed current research progress and predicted the sweetness characteristics and underlying mechanisms of candidate sweet component, thereby establishing a scientific foundation for sweet tea resource and product development. The diversity of sweet tea species furnishes a substantial material basis for constructing differentiated product portfolios. The unambiguous identification of sweet component establishes scientific benchmarks for raw material standardization and quality assurance. Furthermore, this review highlights the health benefits and molecular mechanisms by which sweet tea constituents combat chronic metabolic diseases, with particular emphasis on existing clinical trials investigating sweet tea in metabolic disease management. This work aims to identify synthetic biology targets for the biomanufacturing of natural sweeteners and to inform future clinical management strategies and sustainable development of sweet tea resources.

## 2 Methods

### 2.1 Literature collection

**Figure 1:**
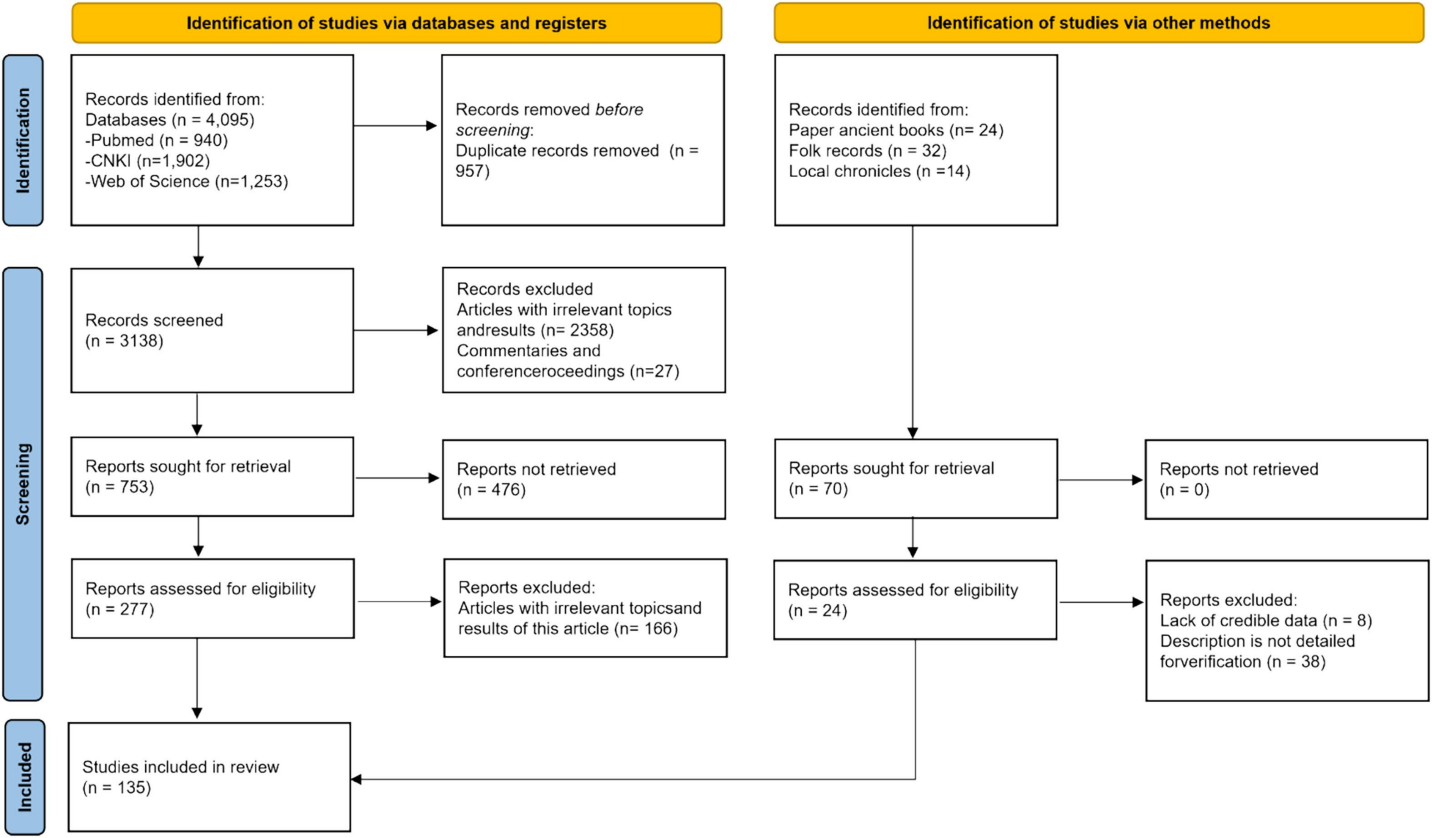
The literature search process (PRISMA, 2020 flow diagram).

Using sweet tea as the keyword, systematic literature (from 1959 to 2025) search was carried out through Chinese medical texts such as *Chinese Botanical Records*, *Dictionary of Chinese Medicine*, *National Collection of Chinese Herbs*, *Dictionary of Medicinal Plants*, *Compendium of Materia Medica* and *Chinese Materia Medica*, as well as databases such as China National Knowledge Infrastructure, Wanfang Database, Web of Science, SciFinder and PubMed, and filtered sweet-tea-irrelevant literature on sugars and synthetic sweeteners. The plant species, geographical distribution, life-form, sweet parts, harvesting, processing and individual sweet components of sweet tea were collected, and the health benefits and molecular mechanisms of sweet components on chronic metabolic diseases were summarized, to provide useful reference for the resource utilization and product development of sweet tea, and to broaden new insights for tea beverages mediated-prevention and treatment of chronic metabolic diseases. A flowchart detailing our screening strategy is provided in Figure 1.

### 2.2 Natural sweet components collation

The sweetness of collected sweet components was collated through China National Knowledge Infrastructure, SciFinder, PubChem database, and the chemical structural similarity of them was also compared with sucrose. The sweetness of them was evaluated by using the sweetness of 2% sucrose as a benchmark and was sweetened through the chemsweet sweetness prediction website^1^ for sweetness prediction, to provide useful reference for the exploitation and utilization of natural sweeteners.

### 2.3 Bibliometric analysis of sweet tea

After using China National Knowledge Infrastructure, Web of Science and other databases as data sources, and filtering irrelevant keywords as tea plants (*Camellia sinensis* L.), fermented teas (including green tea, black tea, white tea, yellow tea, etc.), and flower teas (including jasmine tea, chrysanthemum tea, rose tea, etc.), VOSviewer software was employed to analyze the annual number of publications and keywords. A visual map was created to gather the plant species, sweet components, and health benefits of sweet tea, to analyze the current research hotspots and forecast the future development trends of sweet tea.

### 2.4 Defining the functional sweet tea concept

Based on systematic literature analysis, we defined the scientific concept of functional sweet tea. The deficiencies and limitations of the current sweet tea area were discussed and corresponding strategies were proposed to illuminate future development.

## 3 Species, resource, distribution of functional sweet tea and their harvesting and processing

### 3.1 Diversified functional sweet tea species and their sweet parts

**Figure 2:**
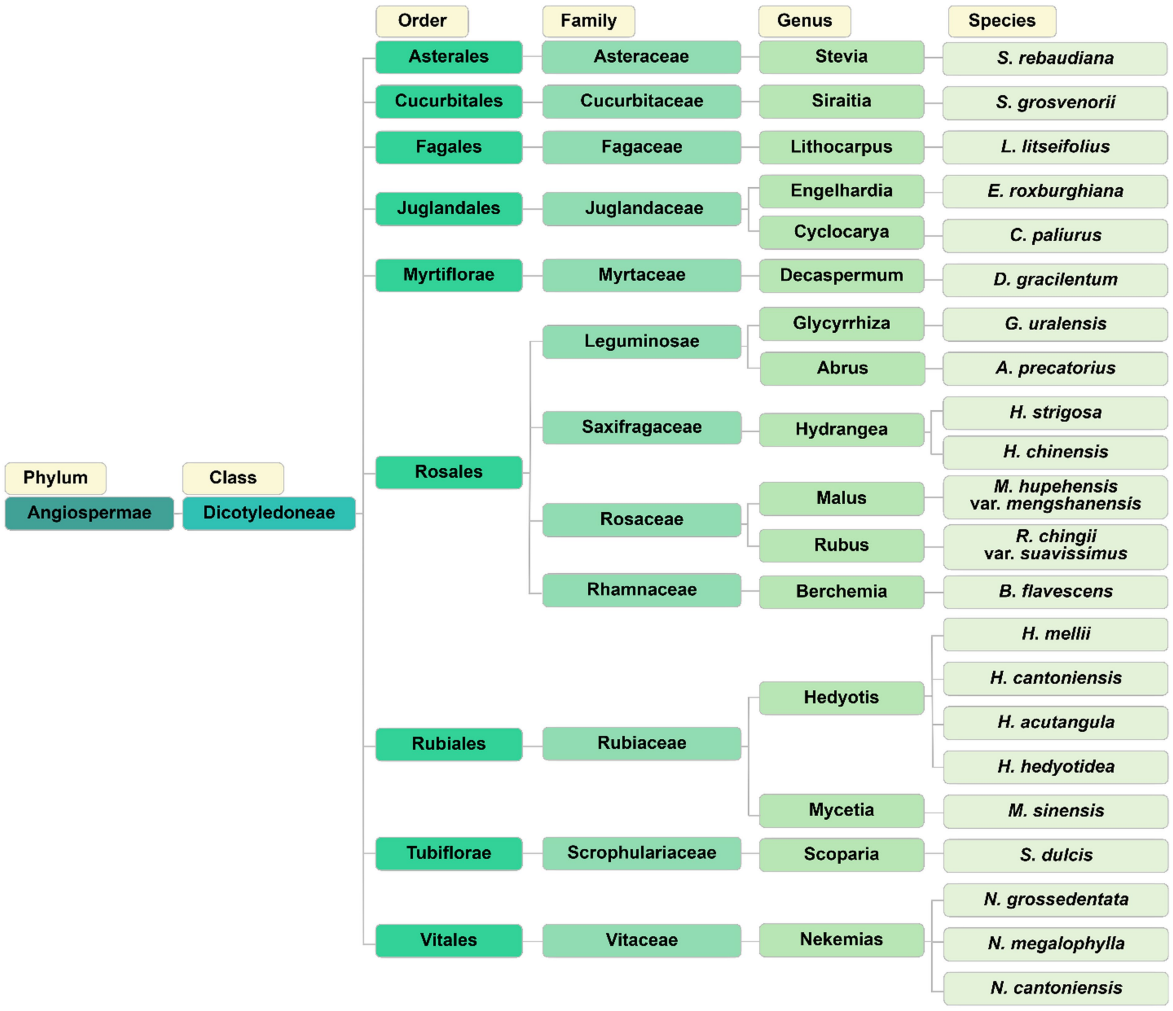
Clustering analysis of functional sweet tea species in a plant taxonomic perspective.

For the first time, Chinese sweet tea species and resources have been systematically collated. Twenty two species of plants commonly known as “sweet tea” in Chinese folklore have been summarized, encompassing 12 families, 16 genera, and 22 species, based on literature analysis and field research. These 22 species belong to the Dicotyledoneae class and 12 families (Figure 2). The family of Rubiaceae has 5 sweet tea species, and *Mycetia sinensis* (Hemsl.) Craib (abbreviated as *M. sinensis* in the following text, following the same pattern) belongs to *Mycetia* genus, *Hedyotis cantoniensis* F. C. How ex W. C. Ko (*H. cantoniensis*), *Hedyotis mellii* Tutcher (*H. mellii*), *Hedyotis hedyotidea* (DC.) Merr. (*H. hedyotidea*), *Hedyotis acutangular* Champ. ex Benth (*H. acutangula*) belong to *Hedyotis* genus. In turn, Vitaceae family includes 3 sweet tea species, namely Nekemias *grossedentata* (Hand.-Mazz.) J. Wen & Z. L. Nie (*N. grossedentata*), *Nekemias megalophylla* (Diels & Gilg) J. Wen & Z. L. Nie (*N. megalophylla*), and *Nekemias cantoniensis* (Hook. & Arn.) J. Wen & Z. L. Nie (*N. cantoniensis*), belonging to *Nekemias* genus. Leguminosae family includes 2 species: *Glycyrrhiza uralensis* Fisch. (*G. uralensis*) and *Abrus precatorius* L. (*A. precatorius*). Saxifragaceae family includes 2 species: *Hydrangea strigosa* Rehder (*H. strigosa*) and *Hydrangea chinensis* Maxim (*H. chinensis*), both of whom belong to *Hydrangea* genus. Rosaceae family includes 2 species: *Rubus chingii* var. *suavissimus* (S. Lee) L. T. Lu (R. *chingii* var. *suavissimus*) and *Malus hupehensis* var. *mengshanensis* G. Z. Qian & W. H. Shao (*M. hupehensis* var. *mengshanensis*). Juglandaceae family includes 2 species: *Cyclocarya paliurus* (Batalin) Iljinsk. (*C. paliurus*) and *Engelhardia roxburghiana* Wall. (*E. roxburghiana*). Other sweet tea species include *Berchemia flavescens* (Wall.) Brongn. (*B. flavescens*) in Rhamnaceae, *Lithocarpus litseifolius* (Hance) Chun (*L. litseifolius*) in Fagaceae, *Siraitia grosvenorii* (Swingle) C. Jeffrey ex A. M. Lu & Zhi Y. Zhang (*S. grosvenorii*) in Cucurbitaceae, *Stevia rebaudiana* (Bertoni) Bertoni (*S. rebaudiana*) in Asteraceae, *Scoparia dulcis L*. (*S. dulcis*) in Vitaceae, and *Decaspermum gracilentum* (Hance) Merr. & L. M. Perry (*D. gracilentum*) in Myrtaceae. In brief, the above analyses showed the diversification of sweet tea species in China (Figure 2).

The natural resources of sweet tea are abundant and diverse in Asia, especially in China, which also contributes to the development of healthy tea beverages. The sweet parts of sweet tea majorly include plant leaves, branches, stems, roots, fruits or whole plants, of which the leaves and the whole plants are most used. The sweet parts of 10 species are derived from their leaves, including arbors like *E. roxburghiana*, *C. paliurus*, *L. litseifolius*, *M. hupehensis* var. *mengshanensis*, and *D. gracilentum*; vines like *A. precatorius* and *R. chingii* var. *suavissimus*; shrubs like *H. strigosa* and *H. chinensis*; and the perennial herb *S. rebaudiana*. Seven species use the whole plants as the sweet part, including vines like *N. grossedentata*, *N. megalophylla*, and *N. cantoniensis*, and the shrub *M. sinensis*. *G. uralensis*, *B. flavescens*, and *S. dulcis* have sweet parts in their roots or stems, while *S. grosvenorii* has sweet parts in its fruit.

### 3.2 Geographical distribution of functional sweet tea species and their life form

**Figure 3:**
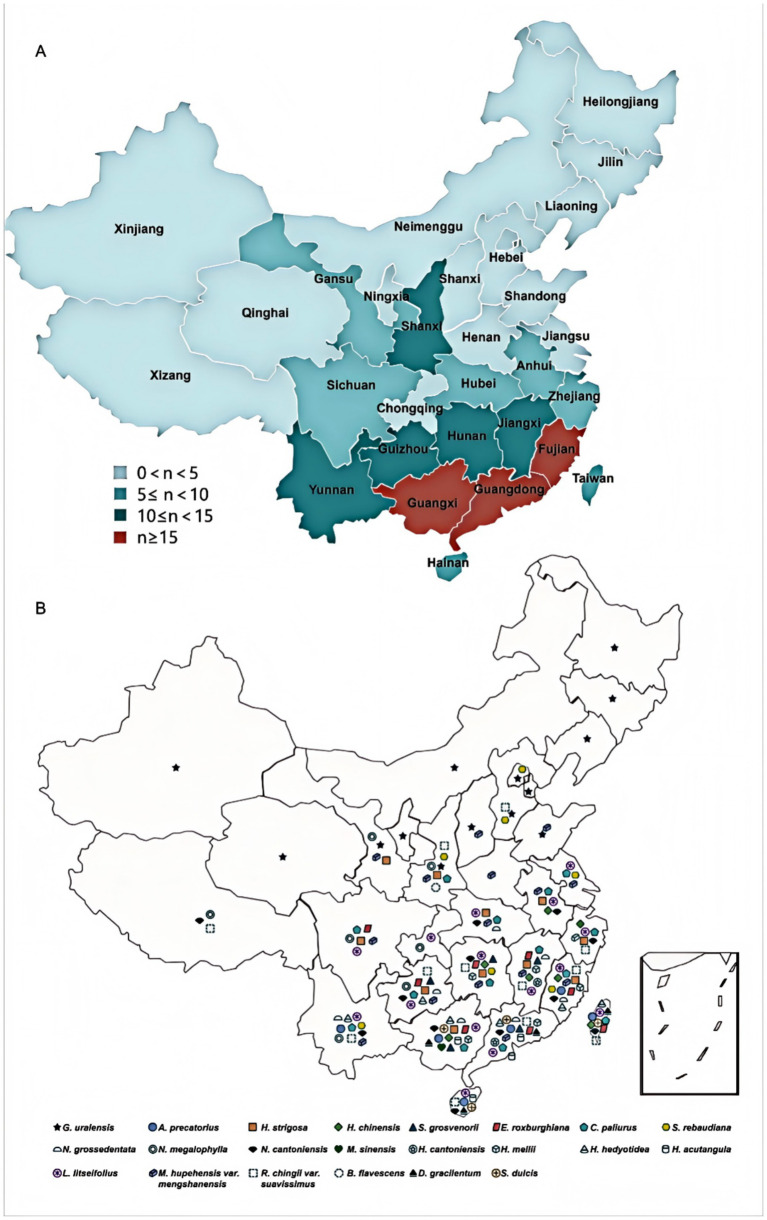
Geographical distribution of functional sweet tea. **(A)** General distributions of sweet in China; **(B)** Specific distribution of each species of sweet tea (different symbols represent different species).

In China, sweet tea exhibits geographic and provenance attributes. Most sweet tea plants are predominantly distributed in Southern region of the Yangtze River in China, with species diversity increasing progressively southward. There are 7 provinces with more than 10 sweet tea species, especially Guangxi Province distributes 18 species of sweet tea. For instance, *R. chingii* var. *suavissimus* is commonly referred to as Guangxi sweet tea due to its widespread recognition in the region. *M. sinensis* is known as Longzhou sweet tea, and *E. roxburghiana* is known as Guiping sweet tea, because these two sweet tea species are consumed and used medicinally by the Yao people and Zhuang people, who are minorities in Longzhou and Guiping in Guangxi province. Secondly, Guangdong and Fujian Provinces also have 16 and 15 species of sweet tea, respectively. Since the cold and harsh natural environment, sweet tea species are almost hard to found in northeast China and the Qinghai-Tibet region, with only *G. uralensis* distributed in the northeast China and N. cantoniensis in Tibet. The above regional analyses suggest that sweet tea may prefer to grow in natural environments with high heat and lots of rainfall (Figure 3).

Most sweet tea species have a wide vertical distribution span. For example, *N. megalophylla* is suitable for living in mountainside scrub or valley open forests at an altitude of 1,300–1,950 m, while *N. cantoniensis* is found in valley forests or hillside shrublands at an altitude of 100–850 m. The above altitude analyses suggest that Chinese sweet tea may prefer to grow in low altitude natural environments.

According to plant life forms in the Vegetation of China, 22 sweet tea species can be mainly divided into 16 woody plants and 6 herbaceous plants. The woody sweet tea includes 6 shrubs, 5 vines, and 5 arbors. *B. flavescens*, *H. strigose*, *H. chinensis*, *M. sinensis*, *H. cantoniensis* and *H. hedyotidea* are shrub plants. *R. chingii* var. *suavissimus*, *A. precatorius*, *N. grossedentata*, *N. megalophylla* and *N. cantoniensis* are vine plants, with the latter three commonly known as sweet tea vines. *M. hupehensis* var. *mengshanensis*, *L. litseifolius*, *D. gracilentum*, *E. roxburghiana* and *C. paliurus* are arbor plants, which *C. paliurus* and *D. gracilentum* are, respectively, referred to as sweet tea tree and sweet tea wood in Chinese folk. In addition, all sweet tea herbaceous plants are perennial, including *G. uralensis*, *S. rebaudiana*, *H. mellii*, *H. acutangular*, *S. dulcis* and *S. grosvenorii*, with *G. uralensis*, *S. dulcis* and *S. rebaudiana* commonly known as sweet tea grass.

### 3.3 Harvesting and processing methods of functional sweet tea

Most sweet tea products are usually processed through conventional drying or dehydration methods following harvest and initial purification. For instance, *L. litseifolius* tea is produced from spring-to-summer harvested young leaves, processed using traditional methods including steaming, pan-firing, and roasting. During processing, stringent control of temperature and duration is essential to prevent thermolabile sweet components (e.g., steviol glycosides, mogrosides) from undergoing hydrolysis, which would result in the loss of sweetness under prolonged high-temperature conditions. Before steaming, sterilization and enzyme inactivation treatment (sha-qing in Chinese) is employed to prevent endogenous glycosidases from cleaving the glycosidic bonds of sweet molecules, thereby preserving sweetness. The resulting infusion exhibits an emerald green hue, refreshing character, and well-balanced aromatic profile with floral and mellow notes. Notably, it produces a persistent sweet aftertaste that endures post-consumption (6).

Alternatively, sweet tea for leaves can be processed according to the green tea production manner. This involves sequential steps including withering, pan-firing, rolling, and final drying. The processed product demonstrates characteristic tightly rolled morphology, vibrant green coloration, and distinct spiral granulation. Furthermore, bioactive constituents may be concentrated using advanced extraction technologies to produce instant sweet tea powder formulations (7).

## 4 Application of functional sweet tea

### 4.1 Regulatory status of functional sweet tea and its sweet components

The therapeutic potential of medicine-food homologous products has gained increasing recognition. Sweet tea serves both as a widely consumed functional beverage in traditional medicine systems and as an ideal raw material for sugar substitutes and nutraceuticals. Various countries have established regulatory frameworks governing different sweet tea species and their bioactive sweet components.

**Figure 4:**
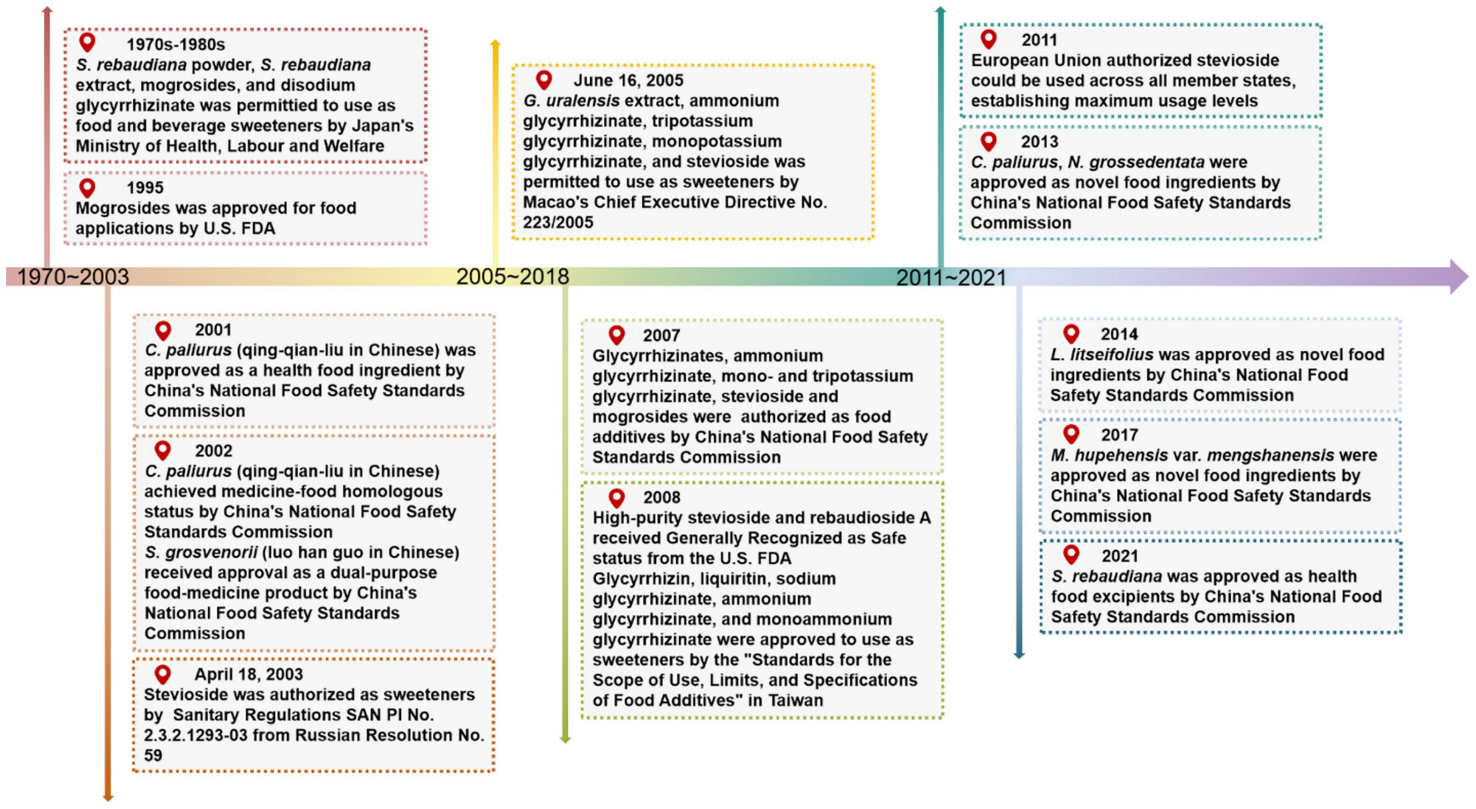
Regulatory status of functional sweet tea and its sweet components.

In 1995, the U.S. FDA approved mogrosides for food applications. Subsequently, regulatory agencies including Singapore’s Agri-Food and Veterinary Authority and Food Standards Australia New Zealand progressively authorized mogrosides as food additives. During the 1970s–1980s, Japan’s Ministry of Health, Labor and Welfare promulgated the Food Sanitation Law Implementation Regulations, permitting the use of *S. rebaudiana* powder, *S. rebaudiana* extract, mogrosides, and disodium glycyrrhizinate as food and beverage sweeteners. In 2001, *C. paliurus* (qing-qian-liu in Chinese) was approved as a health food ingredient by China’s National Food Safety Standardization Committee, achieving medicine-food homologous status in 2002 (8). *S. grosvenorii* (luo-han-guo in Chinese) similarly received approval as a dual-purpose food-medicine product in 2002 (8). On April 18, 2003, Russian Resolution No. 59 approved Sanitary Regulations SAN PI No. 2.3.2.1293-03, authorizing stevioside as sweeteners. On June 16, 2005, Macao’s Chief Executive promulgated Directive No. 223/2005, establishing food additive standards that permitted the use of *G. uralensis* extract, ammonium glycyrrhizinate, tripotassium glycyrrhizinate, monopotassium glycyrrhizinate, and stevioside as sweeteners. In 2007, China’s National Food Additive Standardization Committee authorized glycyrrhizinates, ammonium glycyrrhizinate, mono- and tripotassium glycyrrhizinate, stevioside and mogrosides as food additives. In 2008, high-purity stevioside and rebaudioside A received Generally Recognized as Safe status from the U.S. FDA. On November 20, 2008, Taiwan issued the “Standards for the Scope of Use, Limits, and Specifications of Food Additives,” approving sweeteners included glycyrrhizin, liquiritin, sodium glycyrrhizinate, ammonium glycyrrhizinate, and monoammonium glycyrrhizinate. In 2011, the European Union authorized stevioside use across all member states, establishing maximum usage levels. Recently, *C. paliurus*, *N. grossedentata*, *L. litseifolius* and *M. hupehensis* var. *mengshanensis* were approved as novel food ingredients in 2013 (9), 2013 (9), 2014 (10), and 2017 (11), respectively. In 2021, *S. rebaudiana* was approved as health food excipients under China’s National Food Safety Standards Commission (12). Furthermore, trilobatin, phyllodulcin, and rubusoside have been progressively regulated as flavoring agents or food additives in multiple jurisdictions. For example, rubusoside is primarily utilized as a flavoring agent and holds FEMA GRAS status (FEMA #4717, 4800, 4814) (13) (Figure 4).

### 4.2 Product development and applications of functional sweet tea

Sweet tea and its natural sweet components have gained substantial utilization in premium beverages, functional foods, and nutraceutical products. Within the global sugar reduction initiative, sweet tea assumes increasingly significant importance with growing consumer acceptance. Most sweet tea are primarily consumed as infusions or processed into herbal and sweetened tea beverages, such as the combination of *C. paliurus and Morus alba* L. leaves or *Dendrobium nobile* Lindl. as hypoglycemic tea, which can be used to assist in regulating blood sugar levels (14). In Japan, Kaneka’s glycyrrhizin from *G. uralensis* can slow down fat synthesis and promote fat metabolism. Regulatory-approved functional ingredients derived from sweet tea can be further processed to develop health-oriented products including yogurt, biscuits, canned goods, beer, and confectionery (13, 15, 16). For instance, partial substitution of sucrose with mogrosides in canned citrus products reduces production costs while enhancing sensory profiles. These products are particularly suitable for individuals with cardiovascular diseases, obesity, and diabetes mellitus, while remaining appropriate for consumption across all age demographics (13). Owing to its demonstrated physiological functionalities, sweet tea extracts are also employed in developing skincare products, dietary supplements, and pharmaceutical formulations, including facial cleansers, toners, moisturizing serums, oral mucosal patches, anti-allergenic preparations, and Chinese patent medicine granules (17). For example, *Glycyrrhiza glabra* licorice system restoration from Herb Pharm is a nutritional dietary supplement to promote body repair; DHM (which contains higher purity dihydromyricetin) from Nutricost can be used as an alcohol detoxification health supplement or dietary supplement.

Additionally, high-intensity sweet components from sweet tea can be blended with lower-potency sweetening agents to enhance taste perception, broaden application scope, and meet diverse consumer preferences. For example, sweetener blends incorporating mogrosides, glycyrrhizin, glucosyl stevioside, xylitol, and erythritol exhibit pronounced initial, middle, and lingering sweetness profiles with extended duration, making them particularly suitable for areca nut products and chewing gum applications (18). However, apart from commonly used natural sweeteners such as glycyrrhizin, mogrosides, and steviol glycosides, the development of related products from other natural sweeteners is still at the extract stage. Future efforts should prioritize fundamental research on sweet tea, leveraging advanced technologies in food processing and biotechnology alongside methodological innovations to transform resource advantages into commercial applications and foster innovative development of next-generation sweet tea products.

## 5 Functional sweet components and prediction of their sweetness

**Figure 5:**
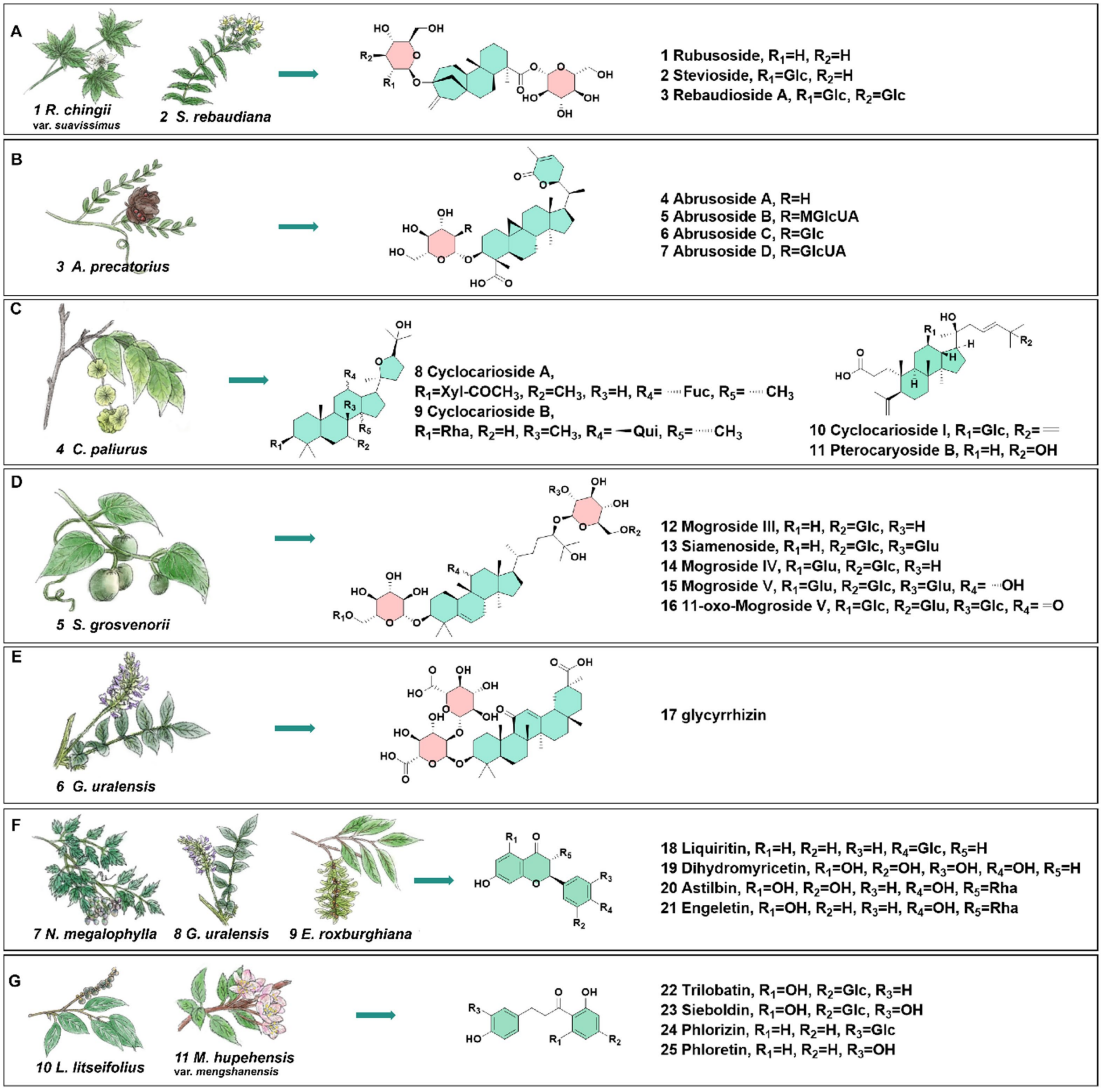
Terpenoids and flavonoids in sweet tea. **(A)** Rubusoside (compound 1) from *R. chingii* var. *suavissimus* (plant 1) and Stevioside, Rebaudioside A (compounds 2–3) from *S. rebaudiana* (plant 2) as *ent*-kaurane tetracyclic diterpenes; **(B)** Abrusosides A–D (compounds 4–7) from *A. precatorius* (plant 3) as cycloartane-type triterpenoids; **(C)** Cyclocariosides A-B, Qingqianliutianosides A and C, Cyclocarioside I, Pterocaryoside B (compounds 8–13) from *C. paliurus* (plant 4) as dammarane-type triterpenoids; **(D)** Mogrosides III–V, 11-oxo-Mogroside V, Siamenoside I (compounds 14–18) from *S. grosvenorii* (plant 5) as cucurbitane-type triterpenoids; **(E)** Glycyrrhizin (compound 19) from *G. uralensis* (plant 6) as oleanane-type pentacyclic triterpenoid; **(F)** Liquiritin, Dihydromyricetin, Astilbin, and Engeletin (compounds 20–23) from *N. grossedentata*, *G. uralensis*, *E. roxburghiana* (plants 7–9) as dihydroflavones; **(G)** Trilobatin, Sieboldin, Phlorizin, Phloretin (compounds 24–27) from *L. litseifolius* and *M. hupehensis* var. *mengshanensis* (plants 10–11) as dihydrochalcones.

Current screening methodologies for sweet components primarily integrate sensory evaluation and metabonomics. However, the identity and composition of sweet components in most sweet tea species remain inadequately characterized, and the molecular mechanisms underlying their sweet effects are poorly understood. Based on documentation analysis, we have unearthed 27 naturally sweet components from various sweet tea species, which can be categorized into 19 terpenoids and 8 flavonoids, exhibiting 50–800 times the sweetness intensity of sucrose (3, 4, 19–26) (Table 1, Figure 5). The 19 terpenoids majorly include 3 tetracyclic diterpenes, 15 tetracyclic triterpenes, and 1 pentacyclic triterpene. The 8 flavonoid-derived sweeteners constitute 4 dihydroflavones and 4 dihydrochalcones.

**Table 1:** Sweet parts and components in different species of sweet tea and their sweetness predictions.

Number	Sweet tea species	Sweet parts	Sweet components	Component type	Sweetness degree	Structural similarity to sucrose/%	Sweetness prediction*	Reference
1	*G. uralensis*	Roots/Stems	Liquiritin	Dihydroflavone	200–800	32.97	4.35	(24)
2	*E. roxburghiana*	Leaves	Astilbin	Dihydroflavone		30.51	3.85	(25)
			Engeletin			31.25	3.79	
3	*N. grossedentata*	Whole Herbs	Dihydromyricetin	Dihydroflavone		18.44	3.59	(26)
4	*N. megalophylla*							
5	*N. cantoniensis*							
6	*L. litseifolius*	Leaves	Trilobatin	Dihydrochalcones	300	37.58	3.67	(4)
			Phlorizin		300	35.99	3.71	
			Sieboldin			36.79	3.73	
7	*M. hupehensis* var. *mengshanensis*	Leaves	Phlorizin	Dihydrochalcones	300	35.99	3.71	(4)
			Phloretin		300	10.44	4.43	
8	*S. rebaudiana*	Leaves	Stevioside	Tetracyclic Diterpenes	300	42.67	4.21	(19)
			Rebaudioside A		450	42.38	4.43	
9	*R. chingii* var. *suavissimus*	Leaves /Branches	Rubusoside	Tetracyclic Diterpenes	300	43.16	4.02	(3)
10	*A. precatorius*	Leaves	Abrusoside A	Tetracyclic Triterpenes	30	42.91	3.5	(20)
			Abrusoside B		100	39.6	4	
			Abrusoside C		50	42.67	3.73	
			Abrusoside D		75	39.96	3.89	
11	*S. grosvenorii*	Fruits	Mogroside V	Tetracyclic Triterpenes	378	45.52	4.55	(20)
			Mogroside III		195	45.51	4.35	
			Mogroside IV		300	45.52	4.53	
			11-oxo-mogroside V		68	43.2	4.67	
			Siamenoside I		465	45.52	4.7	
12	*C. paliurus*	Leaves	Cyclocarioside A	Tetracyclic Triterpenes	200	53.38	4.29	(21)
			Cyclocarioside B		200	54.19	4.02	
			Qingqianliutianosides A		100	57.38	4.02	(22)
			Qingqianliutianosides C		125	51.15	4.22	
			Cyclocarioside I		250	48.48	3.93	(21)
			Pterocaryoside B		100	43.93	3.92	
13	*G. uralensis*	Roots/Stems	Glycyrrhizin	Pntacyclic Titerpenes	70–300	41.06	4.15	(23)

*The website collected molecules with sweetness potency data from databases and literature, including the molecular structures and their sweetness multiplicities relative to sucrose. The molecules were examined and corrected for CAS number and name in the PubChem database, and the same molecule with multiple sweetness potency values was de-duplicated. The final dataset consisted of molecules and their corresponding sweetness potency value, and the values were converted to a logarithmic scale as the target variable for model prediction, i.e., logSw. The higher the logSw value, the sweeter the molecule (130).

### 5.1 Sweet terpenoids

Sweet terpenoids in sweet tea can be divided into 3 tetracyclic diterpenes, 15 tetracyclic triterpenes, and 1 pentacyclic triterpene. Rubusoside (compound 1) from *R. chingii* var. *suavissimus*, stevioside and rebaudioside A (compounds 2–3) from *S. rebaudiana* are tetracyclic diterpenes (3, 19). Regarding triterpenoids, abrusosides A-D (compounds 4–7) from *A. precatorius* are classified as cycloartane-type triterpenoids (20), while cyclocariosides A-B, Qingqianliutianosides A and C, and pterocaryoside B (compounds 8–13) from *C. paliurus* represent dammarane-type triterpenoids (21, 22). Additionally, mogrosides III-V, 11-oxo-mogroside V, and siamenoside I (compounds 14–18) from *S. grosvenorii* are categorized as cucurbitane-type triterpenoids (20). Moreover, glycyrrhizin (compound 19) from *G. uralensis* is classified as an oleanane-type pentacyclic triterpenoid (23). Meanwhile, a web-based sweetness prediction platform was employed to compare the structural similarity of selected sweet components to that of sucrose, which to predict their relative sweetness and to identify structural features potentially influencing the sweetness intensity of these non-caloric natural sweeteners.

#### 5.1.1 Tetracyclic diterpenoid family

Rubusoside, isolated from *R. chingii* var. *suavissimus*, along with stevioside and rebaudiosides A-E isolated from *S. rebaudiana*, belongs to the *ent*-kaurane tetracyclic diterpenoid family. Among them, rubusoside exhibits the highest content (2–5.5% of total plants) and demonstrates significant bioactivities, including anti-allergy, anti-inflammatory, caries prevention, and promotion of glucose and lipid metabolism (27). The structural similarity between rubusoside and sucrose is 43.16%, with a sweetness approximately 300 times that of sucrose (3), yielding a predicted sweetness value of 4.02. At low concentrations, rubusoside imparts a refreshing sweetness with minimal bitter and astringent aftertaste. Higher concentrations yield more pronounced bitterness, though oral adaptation leads to enhanced sweetness perception and a distinctive flavor profile.

**Figure 6:**
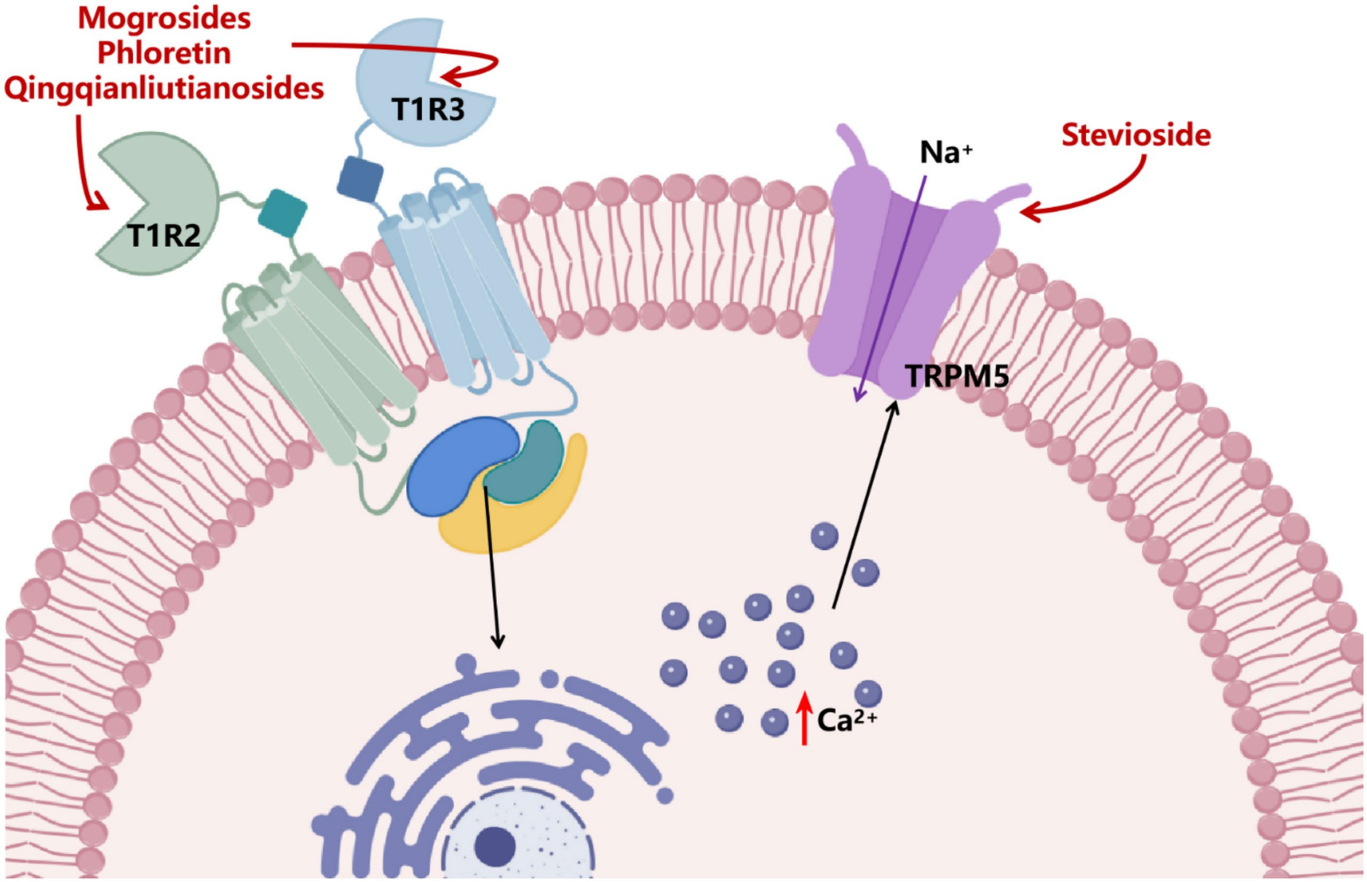
Sweet mechanism by sweet components of sweet tea. T1R2, taste receptor family 1 subunit 2; T1R3, taste receptor family 1 subunit 3; TRPM5, transient receptor potential cation channel subfamily M member 5. T1R2 and T1R3 are the main sweet taste receptors, which can bind to all known types of sweet stimuli to trigger the release of calcium from the endoplasmic reticulum, thereby causing the opening of the monovalent cation-selective TRPM5 channel, which allows sodium ions to flow in, leading to depolarization of the membrane. Mogrosides, phlorizin, and Qingqianliutianosides may exert the sweetening effect through the taste receptor family 1 subunit 2/3 receptors. Stevioside does not directly activate sweet taste receptors but enhances Ca^2+^-dependent channel activity via the Ca^2+^/calmodulin-dependent protein kinase II pathway.

Steviol glycosides, a class of tetracyclic diterpenoids, which comprise at least 9 sweet components in *S. rebaudiana*, has received Generally Recognized as Safe status from the U.S. Food and Drug Administration (FDA). Stevioside and rebaudioside A constitute the predominant glycosides (comprising >80% total content), with rebaudioside A exhibiting the highest relative sweetness potency (450 times that of sucrose) (19). Steviol glycosides as diterpenoid glycosides, possess a molecular structure that diverge significantly from that of sucrose. Consequently, their mechanism of sweetness perception may also differ from the canonical pathway associated with sucrose. Notably, steviol glycosides may not directly activate sweet taste receptors but enhance Ca^2+^-dependent channel activity via the Ca^2+^/calmodulin-dependent protein kinase II pathway (Figure 6). This mechanism potentiates transient receptor potential cation channel subfamily M member 5 ion channel activity, augmenting glucose-induced insulin secretion in pancreatic islets. Therefore, it enhances peripheral taste reactivity in type II taste cells, increases sweetness sensitivity, and attenuates diabetes progression (28).

#### 5.1.2 Tetracyclic triterpene family

Mogrosides III-V, 11-oxo-mogroside V, and siamenoside I from *S. grosvenorii*, classified as cucurbitane-type triterpenoids, are the most common isoprenoid sweeteners approved by the U.S. FDA. Structure–activity relationship studies indicate that the sweetness intensity of mogrosides is critically influenced by the number, positional arrangement, and stereochemistry of glucose moieties, as well as the configuration of glycosidic linkages. A threshold of four glucose residues serves as a critical determinant: molecules containing more than four glucose units demonstrate enhanced sweetness potency, whereas those with fewer exhibit bitter characteristics (29, 30). Glycosylation at the C-3 position reduces perceived bitterness, while additional glucose units at C-24 simultaneously enhance sweetness intensity and suppress bitter notes. This structure-dependent taste modulation may originate from steric compatibility with the ligand-binding pockets of human taste receptors (31). Notably, mogroside V is the strongest sweet component in *S. grosvenorii*, exhibiting 45.52% structural similarity with sucrose, a sweetness 378-fold greater than sucrose (21), and a predicted sweetness value of 4.55 with sensory characteristics closely resembling sucrose. Recently, enzymatic glycosylation of mogroside scaffolds via biosynthetic pathways has emerged as a promising strategy for developing novel sweetening agents with optimized sensory profiles (17, 18), suggesting significant potential as an effective natural sweetener (32, 33).

Abrusosides A-D from *A. precatorius*, characterized by a unique three-membered-carbocyclic-ring structure formed at C-9 and C-19, exhibit predicted sweetness values above 3.5, with sweetness levels 30, 100, 50, and 75 times that of sucrose, respectively (20).

Cyclocariosides A-B, the primary sweet components of *C. paliurus*, exhibit over 50.00% structural similarity to sucrose, with predicted sweetness values exceeding 4.0. Furthermore, their sweetness is approximately 200 times that of sucrose (23), classifying them as promising high-sweetness dammarane triterpenoid sweeteners. Qingqianliutianosides A and C have sweetening effects 100 times and 125 times that of sucrose respectively, and have a long-lasting sweet aftertaste. Molecular docking studies have shown that Qingqianliutianosides A and C exert the sweetening effect through the taste receptor family 1 subunit 2/3 receptors Ser239, Ser380, Thr305, Asn381, Thr1, and Val2 (22). Additionally, cyclocarioside I and pterocaryoside B, as A-3,4-seco-dammarane tetracyclic triterpenes from *C. paliurus*, are slightly less sweet than normal dammarane tetracyclic triterpenes from *C. paliurus* with predicted sweetness values exceeding 3.9, while demonstrating higher hypoglycemic activity (34).

#### 5.1.3 Pentacyclic triterpene family

Glycyrrhizin, including its salts (such as mono-potassium glycyrrhizinate, tripotassium glycyrrhizinate, and diammonium glycyrrhizinate), belongs to oleanane-type pentacyclic triterpenoids in *G. uralensis*. Glycyrrhizin exhibits sweetness 50–300 times that of sucrose, with its salts being sweeter than the acid itself (35). Glycyrrhizin exhibits 41.06% structural similarity to sucrose, with a predicted sweetness value of 4.15, suggesting glycyrrhizin may be a widely used natural sweetener with high sweetness. Glycyrrhizin demonstrates both significant sweetening properties and pharmacological activities. Its sweetness manifests gradually and exhibits notable persistence. In beverage applications, glycyrrhizin functions as a dual-purpose additive that enhances sweetness and aromatic profiles while effectively masking undesirable bitter tastes and off-flavors from other ingredients. In thermally processed foods including meat products and baked goods, glycyrrhizin helps preserve color, aroma, and flavor stability by mitigating thermal degradation and charring effects. Furthermore, glycyrrhizin is approved as a food additive by China’s National Health Commission, and diammonium glycyrrhizinate has been approved by China’s National Medical Products Administration as a hepatoprotective drug to prevent drug- or toxicant-induced liver injury and chronic hepatitis (36).

### 5.2 Sweet flavonoids

Flavonoids in sweet tea can be mainly separated into 4 dihydroflavone and 4 dihydrochalcone. Dihydromyricetin (compound 20) from *N. grossedentata*, *N. megalophylla*, and *N. cantoniensis*, liquiritin (compound 21) from *G. uralensis*, astilbin and engeletin (compounds 22–23) from *E. roxburghiana* represent dihydroflavonoids (24–26). The sweet dihydrochalcones include trilobatin, phlorizin and sieboldin (compounds 24–26) from *L. litseifolius*, and phloretin (compound 27, aglycone of phlorizin) from *M. hupehensis* var. *mengshanensis* (4).

#### 5.2.1 Dihydroflavone family

Liquiritin from *G. uralensis* belongs to dihydroflavonoids, which is listed as a novel food ingredient in the European Union, with the sweetness 200–800 times that of sucrose (24) and exhibiting a predicted sweetness value of 4.35. Astilbin and engeletin from *E. roxburghiana* also belong to dihydroflavonoids, exhibiting a predicted sweetness value of 3.85 and 3.79 (25). Dihydromyricetin is the predominant compound of polyphenols in *N. grossedentata*, *N. megalophylla*, and *N. cantoniensis*, as a primary sweet component exhibiting a predicted sweetness value of 3.59 (26).

#### 5.2.2 Dihydrochalcone family

Trilobatin, phlorizin from *L. litseifolius*, and phloretin (aglycone of phlorizin) from *M. hupehensis* var. *mengshanensis*, are recognized as desirable natural dihydrochalcone sweeteners, and their sweetness is approximately 300 times that of sucrose (4). In comparison to sucrose, phlorizin and related dihydrochalcone derivatives possess a unique sweet taste. These compounds demonstrate delayed sweetness onset followed by development of menthol-like sweet aftertaste during prolonged oral exposure (6). Molecular docking analyses indicate that phlorizin interacts with the sweet taste receptor family 1 subunit 3. Synergistic combinations with sucrose enhance sweetness perception, whereas combinations with catechin suppress sweetness. The sucrose-phlorizin synergy may result from enhanced activation of sweet taste receptor cells via strong interactions with taste receptor family 1 subunit 2/3 proteins, subsequently amplifying neural responses in cerebral regions associated with sweet perception (37). Catechin-phlorizin combinations inhibit intracellular Ca^2+^ signaling, mostly due to reduced binding stability between phlorizin and taste receptor family 1 subunit 3 following catechin addition. This leads to altered activation patterns in central and frontal cortical regions, ultimately masking sweetness perception while enhancing bitter notes and negative hedonic responses (38).

## 6 Prevention and treatment of chronic metabolic diseases of functional sweet components

**Figure 7:**
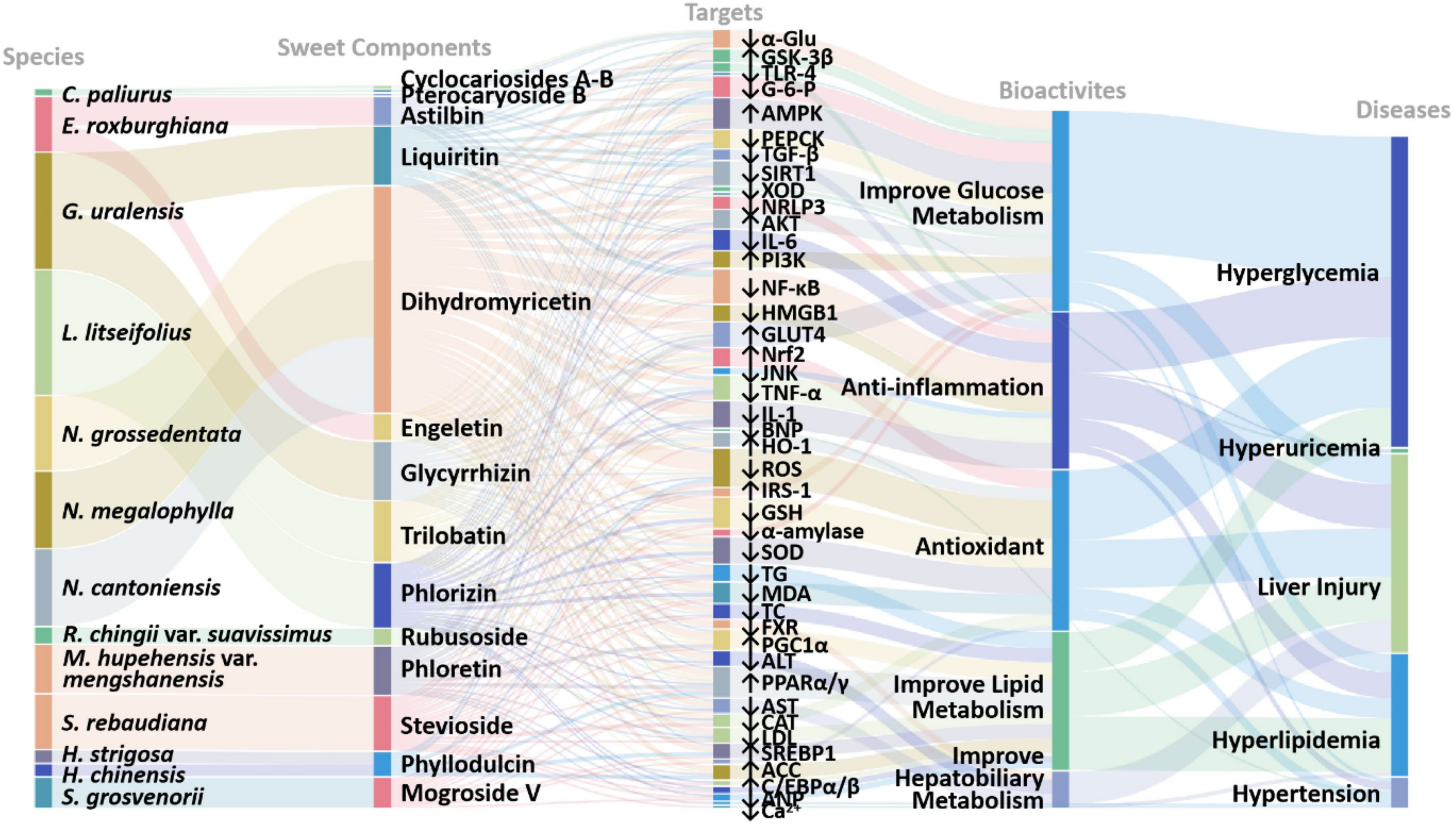
Targets and activities related to improving metabolism diseases effects exerted by functional sweet tea.

**Figure 8:**
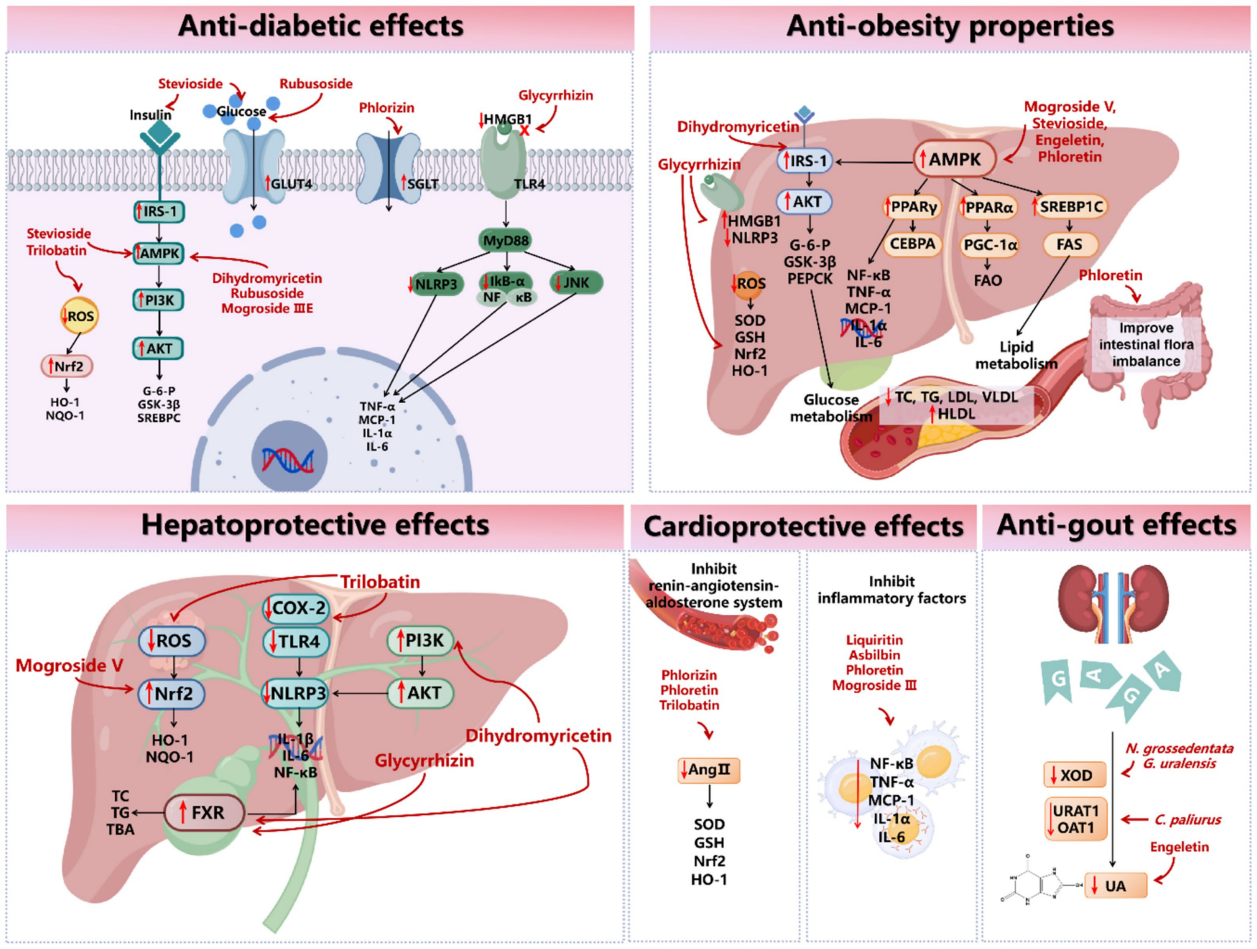
Pathways/targets of metabolism diseases effects by sweet components of sweet tea. IRS-1, insulin receptor substrate 1; AMPK, 5′-adenosine monophosphate-activated protein kinase; PI3K, phosphoinositide 3-kinase; AKT, protein kinase B; GLUT4, glucose transporter 4; SGLT, sodium–glucose cotransporters; ROS, reactive oxygen species; Nrf2, nuclear factor erythroid 2-Related Factor 2; HMGB1, high mobility group box 1; TLR4, toll-like receptor 4; MyD88, myeloid differentiation primary response protein 88; NLRP3, NOD-like receptor thermal protein domain associated protein 3; IkB-α, NF-κB inhibitor alpha; JNK, c-jun N-terminal kinase; NF-κB, nuclear factor-κB; HO-1, oxygenase-1; NQO-1, quinone oxidoreductase 1; G-6-P, glucose-6-phosphate dehydrogenase; GSK-3β, glycogen synthase kinase 3β; SREBPC, sterol regulatory element-binding protein; TNF-α, anti–tumor necrosis factor-α; MCP-1, monocyte chemoattractant protein-1; IL-1α, interleukin 1α; IL-6, interleukin 6; PPAR, peroxisome proliferator-activated receptors; SREBP1C, sterol regulatory element-binding protein 1c; ACC, CEBPA, CCAAT/enhancer-binding protein alpha; PGC-1α, peroxisome proliferator-activated receptor gamma coactivator 1α; FAS, fatty acid synthesis; FAO, fatty acid oxidation; PEPCK, phosphoenolpyruvate carboxykinase; SOD, superoxide dismutase; GSH, glutathione; LDL, low-density lipoproteins; VLDL, very-low-density lipoproteins; HLDL, high-density lipoproteins; COX-2, cyclooxygenase-2; FXR, farnesoid X receptor; TC, total cholesterol; TG, triglyceride; TBA, total bile acid; XOD, xanthine oxidase; UA, urine acid.

Sweet tea showed promising potential for being used in the prevention and treatment of chronic metabolic diseases in China. Chronic metabolic diseases encompassed a serious of chronic illness characterized by disordered metabolism, majorly including hyperglycemia, hyperlipidemia, steatohepatitis, hypertension, and hyperuricemia (39). Fortunately, many functional sweet teas have historically been utilized as traditional and modern clinical medicinal materials for chronic metabolic diseases. Compared with catechins from tea beverages derived from *Camellia sinensis*, specific sweet components from sweet tea, such as glycyrrhizin and mogrosides, were extensively studied for their therapeutic potential against diabetes, obesity, and hepatic disorders. Furthermore, non-sweet major components presented in sweet tea—such as quercetin, kaempferol, rutin, and polyphenol—also exhibited diverse metabolic regulatory activities (40–43). This review will primarily focus on the contributions of the sweet constituents derived from these plants to the management of metabolic diseases, which was discussed in detail (Figures 7, 8).

### 6.1 Anti-diabetic effects

Total extracts, flavonoids, and triterpenes isolated from sweet tea plants, including *A. precatorius* (44, 45), *C. paliurus* (46–48), *S. rebaudiana* (49), and *L. litseifolius* (50, 51), were demonstrated to significantly reduce the blood glucose levels in diabetic animal models. Specific sweet components in sweet tea demonstrated anti-hyperglycemia effects through activation of insulin receptor signaling pathway (51–57), inhibition of sodium-dependent glucose transporters (58), anti-inflammation (36, 48, 59–61) and antioxidant (52, 62, 63) activities (Table 2).

**Table 2:** Pathways/targets of hyperglycemia by sweet components of sweet tea.

Number	Sweet tea species	Sweet components	Disease	Pathways or targets	Reference
1	*G. uralensis*	Glycyrrhizin	Hyperglycemia	↓TC, ↓TG, ↓LDL-C, ↓VLDL-C, ↑HLDL-C, ↓p38MAPK, ↓HMGB1, ↓HMGB2, ↓NLRP3, ↓COX2, ↓TGF-β, ↓TNF-α, ↓IL-1β, ↓IL-6β, ↓TLR2, ↓TLR4, ↓CX43, ↓CXCR1, ↓CXCR4/SDF1, ↓ROS, ↓MDA, ↓SOD, ↑Nrf2, ↓HO-1, ↓GSH, ↓RAGE, ↓11β-HSD	(36, 48, 59–62)
		Liquiritin	Hyperglycemia	↓PEPCK, ↓G-6-P, ↑GSK-3β, ↓α-Glu, ↑AMPK/SIRT1/PGC-1α, ↓TG, ↓TC, ↓LDL-C, ↑HDL-C, ↓ROS	(103)
2	*H. strigosa*	Phyllodulcin	Hyperglycemia	↓G-6-P, ↓PEPCK, ↑SREBP-1, ↑ACC, ↑AP2, ↑FAS, ↑PPAR*γ*, ↓ROS	(131)
	*H. chinensis*				
3	*S. grosvenorii*	Mogroside V	Hyperglycemia	↓SIRT1, ↑PPARα/γ, ↓SREBP1, ↓SCD1, ↓FASN, ↑CPT-1A, ↑ATGL, ↑HSL, ↓ROS, ↓SOD, ↓GSH, ↓NF-κB, ↓p38MAPK, ↓ova-lgE, ↑SQSTM1	(69)
4	*E. roxburghiana*	Astilbin	Hyperglycemia	↓AGEs, ↓G-6-P, ↓SIRT1, ↓TGF-β, ↓CTGF	(132)
		Engeletin	Hyperglycemia	↓α-Glu	(64)
5	*C. paliurus*	Cyclocarioside A	Hyperglycemia	↓α-Glu	(66)
		Cyclocarioside B	Hyperglycemia	↓α-Glu	(66)
		Pterocaryoside B	Hyperglycemia	↓α-Glu	(64)
6	*S. rebaudiana*	Stevioside	Hyperglycemia	↓α-Glu, ↓α-amylase, ↓PDK4, ↑p-AMPK, ↑IR, ↑IRS-1, ↑AKT, ↑TBC1D1, ↑GLUT4, ↑PI3K/AKT, ↑CEBPA, ↑FASN, ↑IκB-α, ↓ IL-1β, ↓IL-6, ↓TNF-α, ↓SOD, ↑CAT, ↓LPO, ↑GSH	(57, 63)
7	*N. grossedentata*	Dihydromyricetin	Hyperglycemia	↑AMPK/mTOR, ↑AMPK-PGC-1α/SIRT3, ↑GLUT1, ↑PI3K/AKT, ↑PLC-CAMKK-AMPK, ↑GLP-1, ↓GSK-3β, ↑MEK/ERK, ↓ROS, ↑GSH, ↑XO, ↓TrkB, ↓HMGB1, ↓NF-κB, ↓IL-1β, ↓IL-6, ↓TNFα, ↓MCP1	(133)
	*N. megalophylla*				
	*N. cantoniensis*				
8	*L. litseifolius*	Trilobatin	Hyperglycemia	↓α-Glu, ↓α-amylase, ↓SGLTs, ↑AKT, ↑GLUT4, ↑p-AMPK, ↓IRS-1, ↑PEPCK, ↓GSK-3β, ↓DPPH, ↑p-ACC, ↓PGC1α, ↓PCK1, ↓G-6-P, ↑GSK-3β, ↓Keap1, ↓CAT, ↓GSH, ↓SOD, ↑Nrf2/ARE, ↓HO-1, ↓NQO-1, ↓GLUT2	(51, 52)
		Phlorizin	Hyperglycemia	↓α-Glu, ↓SGLT1, ↓SGLT2, ↓HNF-1α, ↓CYP2B10, ↓EPHX1, ↑GLUT2, ↑PEPCK, ↑G-6-P, ↑AMPK/PI3K/AKT, ↑IRS-1/PI3K/AKT, ↑GSK3-β, ↑FOXO1, ↓TC, ↓TG, ↓FAS, ↓PAP, ↑HDL-C, ↑CPT, ↓HMGR, ↓ACAT, ↓SOD, ↓MDA, ↓GSH, ↓CAT, ↓ROS, ↑MMP, ↓JNK, ↓IKK-/NFB, ↑CD11c, ↓CD206, ↓MCP-1, TNF-α, IL-1, IL-6, ↓IFN-*γ*	(51, 58)
9	*M. hupehensis* var. *mengshanensis*	Phloretin	Hyperglycemia	↑PI3K/AKT, ↑IRS, ↑PI3K, ↑AS160, ↑GLUT4, ↑PPARγ、PGC1 α, ↑TGF-β, ↓MCP1, ↓BMP2, ↓RANKL	(134)
10	*R. chingii* var*. suavissimus*	Rubusoside	Hyperglycemia	↓α-Glu, ↓α-amylase, ↑p-AMPK, ↑GLUT4, ↓GLUT2, ↓SOD, ↓CAT, ↓GSH, ↓MDA	(68)

#### 6.1.1 Activating insulin signal pathway

Certain sweet components in sweet tea could activate insulin receptor signaling pathways to improve insulin resistance and reducing blood glucose levels (51–57). For instance, engeletin (64), cyclocariosides A-B (65, 66), pterocaryoside B (65, 66), stevioside (67), and rubusoside (68) exhibited inhibitory activity on α-glucosidase to activate the insulin secretion and insulin signaling pathways. Trilobatin and phlorizin could also improve insulin resistance. Insulin receptor substrate and glycogen synthase kinase 3β phosphorylation were inhibited through adenosine monophosphate-activated protein kinase/phosphatidylinositol 3-kinase/protein kinase B pathway to improve insulin resistance, finally reducing the blood glucose levels (51, 52). Stevioside enhanced glucose transporter 4 synthesis through activating the insulin receptor/insulin receptor substrate/protein kinase B/glucose transporter 4 pathway, promoting glucose uptake and oxidation in diabetic muscle (57). Moreover, Mogroside IIIE enhanced adenosine monophosphate-activated protein kinase phosphorylation and inhibited histone deacetylase, which the activity of glucose-6-phosphatase activity was thus suppressed and the blood glucose level was lowered (69).

#### 6.1.2 Inhibiting sodium-dependent glucose transporters

Inhibition of sodium-dependent glucose transporters (SGLTs, including SGLT1 and SGLT2) effectively reduced the process of glucose reabsorption and thus lowered blood glucose. Phlorizin acted as a SGLT inhibitor, reducing glucose reabsorption in the renal tubules and showing anti-hyperglycemia effects (58). Moreover, Phlorizin-derived synthetic antihyperglycemic drugs, including cagliflozin, dagliflozin, empagliflozin, egliflozin, and ergometrine, were approved by the U.S. FDA for clinical use in the treatment of type 2 diabetes mellitus as potent SGLT inhibitors.

#### 6.1.3 Inhibiting high mobility group protein B

Glycyrrhizin was characterized as a high mobility group box 1 (HMGB1) protein inhibitor, which could reduce high mobility group box 1/toll-like receptor 4 (HMGB1/TLR4) receptor binding, in turn downregulating pro-inflammatory mediators such as transcription factor nuclear factor-κB (NF-κB), anti-tumor necrosis factor-*α* (TNF-α), interleukin-1β (IL-1β), interleukin-6 (IL-6), the NOD-like receptor family pyrin domain containing 3 (NLRP3) inflammasome, suppressing immune cell (macrophages, monocytes) activation and inflammatory cytokine release. Terminally, inflammation-mediated insulin signaling impairment was attenuated, glucose uptake and utilization was enhanced, and metabolic dysregulation in high-fat diet/streptozotocin-induced diabetic models was ameliorated, including renal dysfunction, retinopathy, and endothelial impairment (36, 48, 59–61).

#### 6.1.4 Antioxidant stress

The redox imbalance contributed to β-cell dysfunction and modulates insulin resistance-associated signaling pathways, aggravating diabetes symptoms (70). Certain sweet components in sweet sea could inhibit intracellular reactive oxygen species (ROS) generation, protein glycation, glucose autoxidation, and lipid peroxidation to renew redox balance, promoting oxidative stress-mediated pathogenesis of diabetes mellitus and its complications (52, 62, 63). Glycyrrhizin reduced antioxidant enzymes (SOD), catalase, lipid peroxidation and glycation products fructosamine, in turn decreasing hemoglobin-associated free iron and subsequent iron-mediated radical reactions to attenuate streptozotocin-elevated serum fructosamine levels (62). Stevioside demonstrated antihyperglycemic effects in obese murine models by restoring redox homeostasis, downregulating ROS and NO while modulating SOD expression, and terminally ameliorating adipocyte dysfunction and insulin resistance (63). Furthermore, Trilobatin exhibited ROS-scavenging capacity, and upregulated antioxidant enzymes such as catalase, glutathione peroxidase, SOD, meanwhile promoting nuclear factor erythroid 2-related factor 2 (Nrf2)/antioxidant response element pathway by activating nuclear translocation of Nrf2 and upregulating expression of downstream targets like oxygenase-1 (HO-1), quinone oxidoreductase 1, glucose transporter 2, which could significantly repair the morphology of pancreatic islet cells and enhance insulin expression (52).

#### 6.1.5 Improve intestinal flora imbalance

Intestinal flora imbalance critically influences the progression of chronic diseases (71). Mogrosides reduced the relative abundance of *Firmicutes* and *Proteobacteria* and increased the relative abundance of *Bacteroidetes* in the intestines of diabetic mice, ameliorating diabetes through gut microbiota modulation (72).

#### 6.1.6 Related clinic trials for diabetes

Clinical investigations also indicated the significant therapeutic potential of sweet tea [especially *C. paliurus* (73) and *S. dulcis* (74)] in the management of type 2 diabetes mellitus. Fabio (74) assessed the glucose-lowering effect of *S. dulcis*, conducted a 6-month randomized controlled trial on 160 patients with type 2 diabetes mellitus, and results revealed that *S. dulcis* extract powder (300 mg, three times a day) could lead to a reduction of glycated hemoglobin by at least 5.1%, compared with placebo (300 mg, three times a day). Additionally, Peng (73) conducted an 84-day open randomized controlled trial involving 38 type 2 diabetes patients, demonstrating that water extracts of *C. paliurus* (5 mg, three times a day) significantly improved glucose metabolism as glycated hemoglobin levels, blood lipid profiles, and blood pressure.

### 6.2 Anti-obesity properties

The total extracts of *G. uralensis* (75), *S. grosvenorii* (76), *S. rebaudiana* (77, 78), *E. roxburghiana* (79), *C. paliurus* (47, 80–82), and *R. chingii* var. *suavissimus* (83) exhibited significant lipid-lowering effects. Specific sweet components of sweet tea ameliorated hyperlipidemia via activating adenosine monophosphate-activated protein kinase pathway activation (84–88), accelerating lipid metabolism (85, 88, 89), inhibiting inflammatory pathway and oxidative stress (90–93) (Table 3).

**Table 3:** Pathways/targets of hypolipidemic by sweet components by sweet tea.

Number	Sweet tea species	Sweet components	Disease	Pathways or targets	Reference
1	*G. uralensis*	Glycyrrhizin	Hyperlipidemia	↓TG, ↓TC, ↓LDL-C, ↑PPARγ, ↑INSR, ↓PEPCK, ↓G-6-P, ↓STAT17, ↑STAT5, ↓GLUT4, ↓FMN, ↑Nrf2, ↑HO-1, ↓SOD, ↓MDA	(90, 91)
		Liquiritin	Hyperlipidemia	↓FAS, ↓SREBP1, ↓HMGB1, ↓TLR4, ↓NLRP3, ↓IL-1β, ↓NF-κB, ↓α-SMA	(140)
2	*S. grosvenorii*	Mogroside V	Hyperlipidemia	↓SIRT1, ↑p-AMPK, ↑SQSTM1, PPARα/γ, ↓SREBP1, ↓SCD1, ↓FASN, ↑CPT-1A, ↑ATGL, ↑HSL, ↓SOD, ↓GSH, ↓ROS	(84)
3	*E. roxburghiana*	Astilbin	Hyperlipidemia	↓TC, ↓TG, ↓AST, ↓ALT, ↓TBARS, ↑cAMP, ↓LPL, ↑AMPK, ↑SCFA, ↓IL-6, ↑IL-10, ↓TNF-α	(132)
		Engeletin	Hyperlipidemia	↑PPARα, ↑CPT, ↑ACO, ↑ATGL, ↑β3-AR, ↑Nrf2, ↑TFAM, ↑UCP-1, ↑PGC-1α, ↑PRDM16, ↑TMEM26, ↑TBX1, ↑CD137, ↑Cited1, ↑PKA, ↑AMPK	(85)
4	*S. rebaudiana*	Stevioside	Hyperlipidemia	↓TG, ↓TC, ↓LDL-C, ↑HDL-C, ↑PPARα, ↑MAP1LC3β, ↓p62, ↑LAMP1, ↑Beclin 1, ↑AMPK	(86, 89)
5	*N. grossedentata*	Dihydromyricetin	Hyperlipidemia	↓TG, ↓TC, ↓LDL-C, ↑IRF4/PGC-1α, ↑WAT, ↑PI3K/AKT/FoxO3a, ↑p-AMPK, ↑p-IRS-1, ↑p-AKT, ↑GLUT4, ↓SOD, ↑DDAH1, ↓ROS, ↓NF-κB, ↓NLRP3	(87, 141)
	*N. megalophylla*				
	*N. cantoniensis*				
6	*L. litseifolius*	Phlorizin	Hyperlipidemia	↓TC, ↓TG, ↓LDL-C, ↑HDL-C, ↓FAS, ↓AST, ↓ALT, ↓PAP, ↑ApoA-I, ↑CPT, ↓HMGR, ↓ACAT, ↑UCP1, ↑PPARα/γ, ↑C/EBPβ, ↑PRDM16, ↓MDA, ↓GPX, ↓SOD, ↓TNF-α, ↓IL-1, ↓IL-6, ↑Tyk2/STAT3	(142)
7	*M. hupehensis* var. *mengshanensis*	Phloretin	Hyperlipidemia	↓TG, ↓TC, ↓LDL-C, ↑PPARγ, ↑CD36, ↑APETALA2, ↑GLUT4, ↑ACCα, ↑C/EBPα, ↑AMPK, ↓SIRT1/PGC-1α, ↓SIRT3/CYPD	(88)

#### 6.2.1 Activating adenosine monophosphate-activated protein kinase pathway

Adenosine monophosphate-activated protein kinase (AMPK) was a crucial metabolic regulator that promoting cellular lipid metabolism. Several sweet components in sweet tea activated the AMPK pathway, contributing to the reduction of blood lipid levels (84–88). Mogroside V improved liver lipid metabolism imbalance through activating AMPK-dependent pathway, enhancing lipolysis and fatty acid oxidation by upregulating peroxisome proliferators-activated receptor-α, sterol regulatory element-binding protein, and fatty acid synthase expression, thereby reducing lipid accumulation (84). Engeletin activated the β3-adrenergic receptors/AMPK signaling pathway, increasing the expression of metabolic targets related to fat decomposition and oxidation such as levels of uncoupling protein 1, PR domain containing 16, and peroxisome proliferator-activated receptor gamma coactivator 1α (PGC-1α) to stimulate white adipocytes to transform into brown or brown adipocytes, promoting fat metabolism and heat production, terminally reducing fat storage and prevent obesity (85). Stevioside enhanced the phosphorylation of AMPK and acetyl-CoA carboxylase both *in vitro* and *in vivo* by upregulating AMPK signaling, leading to the downregulation of peroxisome proliferators-activated receptor γ (PPARγ), sterol regulatory element-binding protein 1, CCAAT/enhancer binding protein α, and fatty acid synthase (86). Dihydromyricetin interacted with phospholipase C through the Ca^2+^–Ca/calmodulin-dependent protein kinases-AMPK pathway, increasing the protein levels of phosphorylated-AMPK, phosphorylated-insulin receptor substrate, phosphorylated-protein kinase B and phosphorylated-glucose transporter 4. Terminally, the fat accumulation was reduced and insulin resistance in high-fat diet-induced obese mice was improved (87). Phloretin promoted mitochondrial biosynthesis and enhanced mitochondrial function via activation AMPK-dependent silent information regulator 1/PGC-1α signaling pathway, then facilitated fatty acid β-oxidation and ameliorated liver steatosis (88).

#### 6.2.2 Activating peroxisome proliferator-activated receptor

Activation of peroxisome proliferator-activated receptors (PPARs, including PPARα, PPARβ and PPARγ) contributed to obesity intervention. Engeletin could significantly increase the expression of PPARα, activating the expression of metabolic genes related to fat decomposition and oxidation, such as activating PGC-1α, protein kinase A, fatty triglyceride lipase, carnitine palmitoyl transferase, and acyl-coA oxidase, enhancing lipolysis and β-oxidation to mitigate obesity (85). Stevioside ameliorated hepatic steatosis in db/db mice and HepG2 cells through activation of PPARα/PGC-1α-mediated autophagy, reducing hepatic lipid accumulation, enhancing β-oxidation, and improving non-alcoholic fatty liver disease-associated insulin resistance (89). Phloretin enhanced adipocyte differentiation through upregulating CCAAT/enhancer binding protein α and PPARγ, inhibiting the activation of cyclin-dependent kinase-5 and improving glucose and non-esterified fatty acid utilization (88).

#### 6.2.3 Inhibiting inflammatory pathway and oxidative stress

Inflammatory cascades and oxidative stress could exacerbate dyslipidemia and its complications, including atherosclerosis. Glycyrrhizin could ameliorate high-fat diet-induced obesity by reducing hepatic lipid peroxidation, upregulating insulin receptors and activating Nrf2/HO-1 pathway (90). Glycyrrhizin also attenuated lipopolysaccharide/high-fat-diet-induced atherosclerosis by suppressing HMGB1/NLRP3 inflammasome in atherosclerosis (91). Dihydromyricetin could enhance mitophagy, suppressing NF-κB-dependent NLRP3 inflammasome activation and upregulating miR-21-mediated dimethylaminohydrolase 1/asymmetric dimethylarginine/NO signaling, leading to ameliorate TNF-α-induced endothelial dysfunction, inhibit macrophage foam cell formation, and improve lipid profiles, collectively preventing atherosclerosis (92, 93).

#### 6.2.4 Improve intestinal flora imbalance

Trilobatin could remodel gut bacteria, increase *Lactobacillus*, *Prevotella*, CF231, *Bacteroides*, and *Oscillospira*, while decrease *Blautia*, *Allobaculum*, *Phascolarctobacterium*, and *Coprococcus*, reducing the *Firmicutes*/*Bacteroidetes* ratio to exert anti-obesity effects (94). *S. grosvenorii* triterpenoids also improved gut microbiota composition, lowering the *Firmicutes*/*Bacteroidetes* ratio to mitigate obesity (95).

### 6.3 Hepatoprotective effects

The flavonoid part of *C. paliurus* (96, 97) and ethanol extract of *G. uralensis* (98) all have anti-liver injury and fibrosis effects. The hepatoprotective effects of various sweet tea against multiple liver diseases were primarily attributed to its promoting bile acid metabolism (99–102), antioxidant (56, 103, 104) and anti-inflammation (105–107) properties (Table 4).

**Table 4:** Pathways/targets of anti-liver diseases by sweet components of sweet tea.

Number	Sweet tea species	Sweet components	Diseases	Pathways or targets	Reference
1	*G. uralensis*	Glycyrrhizin	Liver Injury	↓AST, ↓ALT, ↓LDH, ↑FXR, ↑BSEP, ↑MRP4, ↑MRP2, ↓p-MLKL, ↓p-RIPK1, ↓p-RIPK3, ↑Caspase 8, ↑c-FLIPL, ↓PI3K/AKT, ↓BECN1, ↓LC3-II/LC3-I, ↓BECN1-Xct, ↓VEGF, ↓P4502E1, ↓TNF-α, ↓TLR4, ↓p-NF-κB, ↓CCL2, ↓CXCL1, ↓IL-6β, ↓IL-3	(99–101)
		Liquiritin	Liver Injury	↓AST, ↓ALT, ↓TBIL, ↓TPAI-2, ↑CYP4A10, ↑CPT1A, ↑PPARα, ↑EHHADH, ↑ACOX1, ↑Nrf2, ↓TLR4/NF-κB, ↓MMP-2, ↑LATS1, ↓YAP, ↓IL-6, ↓IL-8, ↓MPO, ↓TGF-β1/Smad	(135)
2	*S. grosvenorii*	Mogroside V	Liver Injury	↓ALT, ↓AST, ↓MDA, ↓Keap1, ↓ROS, ↑Nrf2, ↑HO-1, ↑GCLM, ↑NQO1, ↓T-AOC	(56)
3	*E. roxburghiana*	Engeletin	Liver Injury	↓ROS, ↑Nrf2, ↓ECM, ↓EMT, ↓α-SMA, ↓PINK1/Parkin, ↑PXR	(136)
		Astilbin	Liver Injury	↑PPARγ, ↓NF-κB	(125)
4	*S. rebaudiana*	Stevioside	Liver Injury	↓MDA, ↓TNF-α, ↓NF-κB, ↓COX2, ↓GST, ↓CAT	(137)
5	*N. grossedentata*	Dihydromyricetin	Liver Injury, Liver Fibrosis	↓AST, ↓ALT, ↑FXR, ↓CYP1E4, ↓CK18, ↓FGF21, ↑PI3K/AKT, ↑AMPK, ↓FoxO1, ↓G-6-P, ↓PEPCK, ↓TC, ↓TG, ↓LDL-C, ↓MDA, ↓1-HNE, ↓SREBP1, ↑ACC, ↓CPT-1α, ↑GLUT1, ↑PPARα, ↓ROS, ↓iNOS, ↓SOD, ↓NLRP3, ↓p38MAPK, ↓JNK, ↑GSH,↓TNF-α, ↓IL-3β, ↓NF-κB	(92, 93, 102, 107)
	*N. megalophylla*				
	*N. cantoniensis*				
6	*L. litseifolius*	Trilobatin	NAFLD, ALD, FHF	↑AMPK, ↑PPARα, ↓NLRP3, ↓NF-kB, ↓Caspase-1, ↓N-GSDMD, ↓CAT, ↓SOD, ↓GSH, ↑Nrf2, ↓YAP, ↑HO-1, ↑NQO1, ↓ROS/TLR4/NLRP3, ↓IL-18, ↓IL-1β	(104–106)
		Phloretin	Liver Fibrosis, NAFLD	↑SIRT1/PGC-1α, ↓GLUT-2, ↓LDHA, ↑FAO	(138, 139)

#### 6.3.1 Activating farnesoid X receptor

Activation of the farnesoid X receptor (FXR) could exert hepatoprotective effects through suppressing cytokine-mediated inflammatory responses and enhancing bile acid metabolism. Glycyrrhizin upregulated FXR expression to suppress CYP3A11 and CYP2E1 activity, enhance biliary bile acid excretion and inhibit NLRP3 inflammasome activation, followed by attenuating hepatocyte necrosis and inflammatory infiltration, ameliorating liver injury and non-alcoholic steatohepatitis (99–101). Dihydromyricetin also demonstrated hepatoprotection through FXR activation and subsequent downregulation of hepatic inflammatory mediators (NLRP3, IL-1β, IL-18) (102).

#### 6.3.2 Activating nuclear factor erythroid 2-related factor 2

Nuclear factor erythroid 2-related factor 2 (Nrf2) was a key transcription factor for enhancing antioxidant capacity and protecting the liver. Liquiritin could facilitate Nrf2 nuclear translocation to upregulate antioxidant gene expression, and promote hepatocyte proliferation through cell cycle modulation, thus mitigating monocrotaline-induced hepatic oxidative stress (103). Mogroside V significantly activated Nrf2 signaling pathway enzymes, increasing the expression of HO-1, quinone oxidoreductase 1, and Glutamate-Cysteine Ligase Modifier Subunit, thereby exerting anti-liver injury effects (56). Trilobatin exerted anti-liver injury effects by activating the yes-associated protein 1/Nrf2 signaling pathway and regulating intestinal flora homeostasis (104).

#### 6.3.3 Inhibiting NOD-like receptor family pyrin domain containing 3

Inhibition of the NOD-like receptor family pyrin domain containing 3 (NLRP3) inflammasome could effectively reduce hepatic inflammatory disorders. Trilobatin inhibited NLRP3 inflammasome and cyclooxygenase-2, in turn attenuates inflammation and demonstrate efficacy in non-alcoholic fatty liver disease and galactosamine/lipopolysaccharide-induced fulminant hepatic failure through inhibition of ROS/TLR4/NLRP3 signal pathway (105, 106). Dihydromyricetin demonstrated ROS-scavenging capacity and phosphatidylinositol 3-kinase/protein kinase B phosphorylation inhibition through NLRP3 pathway modulation to attenuate protein kinase B-dependent NF-κB activation and suppress inducible nitric oxide synthase expression, in turn removing pro-inflammatory cytokine production and significantly alleviating liver injury (107).

#### 6.3.4 Improve intestinal flora imbalance

Liver diseases are closely linked to alterations in the composition and function of intestinal flora imbalance (108). Correlation analyses indicate that the glycyrrhizin-mediated regulation of hepatic total cholesterol and triglyceride levels is associated with decreased relative abundances of Lachnospiraceae, Coriobacteriaceae, Blautia, and Collinsella, alongside increased relative abundances of *Romboutsia* and *Turicibacter* within the gut microbiota. Moreover, the efficacy of glycyrrhizin significantly exceeds that of flavonoids and extract of *G. uralensis* (109).

#### 6.3.5 Related clinic trials for liver diseases

Clinical evidence supports the therapeutic efficacy of glycyrrhizin across multiple hepatic disorders, including non-alcoholic fatty liver disease (110), drug-induced liver injury (111), chronic liver disease (111), hepatitis C (112), and hepatocellular carcinoma (113, 114), which was discussed in detail below.

Based on a 2-month randomized, double-blind, placebo-controlled trial (*n* = 66 non-alcoholic fatty liver disease patients), glycyrrhizin (2 g/day) was founded to significantly reduced serum aspartate aminotransferase (AST) and alanine aminotransferase (ALT) levels while improving albumin concentrations, indicating hepatoprotective effects of glycyrrhizin and alleviating the symptoms of non-alcoholic fatty liver disease (110).

Meng (111) conducted a multicenter, randomized, double-blind trial (*n* = 412 chronic liver disease patients including drug-induced liver injury), evaluating multiple glycyrrhizin intervention over 4 weeks, with ALT, AST and other clinical and biochemical assessments in weeks 2, 4 and follow-up. The result demonstrated that magnesium isoglycyrrhizinate (the active metabolites of glycyrrhizin *in vivo*) significantly reduced ALT/AST levels by 72.22–73.53% efficacy, accompanied by favorable safety profile (1.18–1.85% adverse events). Subsequently, magnesium isoglycyrrhizinate received China’s National Medical Products Administration approval in 2015 for acute drug-induced liver injury, particularly for hepatocellular damage and mixed-liver injury patterns with marked ALT elevation.

Van (112) assessed intravenous glycyrrhizin efficacy in a four-week trial in hepatitis C patients. Compared with placebo, the ALT levels in the patients injected with glycyrrhizin three times a week and the group injected with glycyrrhizin six times a week decreased by an average of 26 and 47% respectively, compared with the initial stage of treatment, demonstrating glycyrrhizin’s dose-dependent efficacy in hepatitis C management. Yasuji (113) retrospectively analyzed the long-term preventive effect of Stronger Neo-Minophagen C (SNMC, glycyrrhizin-containing formulation,) on the chemopreventive potential of hepatocellular carcinoma. It was found that among SNMC-treated patients (treated with a mixture of 300 mg of glycyrrhizinic ammonium salt and 60 μg of sodium capric acid), the incidence of hepatocellular carcinoma in patients with normal ALT levels was lower than that in patients with abnormal ALT levels, which proved that the reduction in the risk of hepatocellular carcinoma in the SNMC group might be partly due to the improvement of ALT levels, with additional benefits in preventing hepatic histological deterioration in chronic hepatitis C. A meta-analysis of 18 clinical trials also confirmed glycyrrhizin’s hepatoprotective effects in post- hepatocellular carcinoma resection patients, demonstrating significant ALT, AST, bilirubin reduction and albumin elevation (114).

### 6.4 Cardioprotective effects

#### 6.4.1 Inhibit renin-angiotensin-aldosterone system

Flavonoids from *L. litseifolius* leaves attenuated hypertension through suppressing plasma renin activity and angiotensin II in the renin-angiotensin-aldosterone system, then inhibiting oxidative stress markers (e.g., NO, SOD, malondialdehyde) (115) (Table 5).

**Table 5:** Pathways/targets of hypotensive and lowering uric acid by sweet components by sweet tea.

Number	Sweet tea species	Sweet components	Disease	Pathways or targets	Reference
1	*G. uralensis*	Liquiritin	Hypertension	↓BNP, ↓CK, ↓CK-MB, ↓LDH, ↓cTnI, ↓SIRT1, ↑AMPK, ↓Mtor, ↓Col-I, ↓Col-III, ↓TGF-β1, ↓MMP-9, ↓α-SMA, ↓CCL5, ↓Bcl-2, ↑BAX, ↓NF-κB	(116, 117)
2	*S. grosvenorii*	Mogroside IIIE	Hypertension	↓TLR88, ↓MyD1, ↓TGF-β, ↓α-SMA, ↓NF-κB	(119)
3	*E. roxburghiana*	Astilbin	Hypertension	↑Nrf2/HO-1, ↓TLR4/NF-κB	(118)
		Engeletin	Hypertension	↑QTc, ↑ERP, ↑APD, ↑Cx2, ↓VF, ↓ROS, ↓SOD, ↓GSH, ↓MDA, ↑Nrf2, ↑HO-1	(143)
4	*S. rebaudiana*	Rebaudioside A	Hypertension	↓Ca2+	(144)
5	*N. grossedentata*	Dihydromyricetin	Hypertension	↓HMGB1, ↓NF-κB	(133)
	*N. megalophylla*				
	*N. cantoniensis*				
6	*M. hupehensis var. mengshanensis*	Phloretin	Hypertension	↓ANP, ↓SIRT1, ↓IL-1, ↓TNF-α	(88)
7	*E. roxburghiana*	Engeletin	Hyperuricemia	↓XOD	(125)
		Astilbin	Hyperuricemia	↓Cith3, ↓NETs, ↓PMNE, ↓MPO, ↓P2Y6R, ↓IL-8/CXCR2	(145)

#### 6.4.2 Inhibit inflammatory factors

Inflammatory mechanisms significantly contributed to the pathophysiology of hypertensive cardiovascular and cerebrovascular disorders. Liquiritin exerted cardioprotection through inhibiting mammalian target of rapamycin complex 1 and NF-κB/p65 phosphorylation to dose-dependently downregulate TNF-α, IL-6 and IL-1β expression, resulted in attenuation of inflammatory cell infiltration, oxidative stress and apoptosis, and amelioration lipopolysaccharide-induced cardiac dysfunction in murine models (116, 117). Phloretin demonstrated cardioprotective effects by inhibiting silent information regulator 1 and subsequently reducing inflammatory mediators such as IL-1β, TNF-α, and atrial natriuretic peptide (88). Astilbin activated Nrf2/HO-1 and inhibited TLR4/NF-κB to mitigate lipopolysaccharide-induced myocardial injury (118). Mogroside IIIE suppressed myocardial inflammatory cytokine release like TLR4, toll-like receptor 88, transforming growth factor-β1, α-smooth muscle actin through inhibition of TLR4/toll-like receptor 88/NF-κB pathway, which attenuated isoproterenol-induced myocardial fibrosis in C57BL/6 mice (119).

#### 6.4.3 Related clinic trials for hypertension

Notably, clinical data indicated that stevioside maintained vasodilation. Hsieh (120) conducted a 2-year experimental study on Chinese patients with mild hypertension, demonstrating that stevioside (500 mg, 3 times a day) significantly reduced diastolic and systolic blood pressure compared to placebo. Furthermore, patients’ quality of life improved without significant adverse reactions post-stevioside administration.

### 6.5 Anti-hyperuricemic effects

Sweet tea and its numerous sweet components could reduce uric acid levels by inhibiting xanthine oxidase (XOD) activity (121–125) and reducing the expression of transporters like URAT1 and NLRP3 (123, 124, 126, 127) (Table 5).

The water extracts of *G. uralensis*, *N. grossedentata* and *C. paliurus* significantly inhibited XOD activity to reduce uric acid production and ameliorate purine metabolism disorders in various hyperuricemic animal models and gouty arthritis model (121–124). By inhibiting hepatic XOD activity, engeletin could dose-dependently reduce toe swelling, serum inflammatory factors, uric acid, and urea nitrogen levels. This inhibition enhanced uric acid excretion, slowed chronic gout progression (125).

The extract of *N. grossedentata* inhibited urate reabsorption through downregulation of URAT1 and GLUT9 transporters, while promoting urate excretion via upregulation of ABCG2 expression, thereby enhancing uric acid transport flux and ultimately achieving synergistic hypouricemic effects (124, 126). The water extract of *C. paliurus* demonstrated significant plasma uric acid reduction and ameliorated renal fibrosis. Plasma metabolomic analysis revealed its capacity to normalize dysregulated purine metabolism through inhibition of hepatic URAT1 and OAT1 transporters (123). Characteristic cyclolanostane triterpenoids from *C. paliurus* significantly downregulated protein expression of IL-1β, caspase-1, pro-IL-1β, pro-caspase-1, and NLRP3 inflammasome components. Furthermore, these compounds suppressed ROS generation, IL-1β production, and NLRP3 inflammasome assembly. Additionally, dammarane-type triterpenoids from *C. paliurus* modulated the phosphorylation ratios of PI3K, AKT, and mTOR signaling pathways. PI3K-AKT–mTOR-dependent autophagy activation effectively mitigated NLRP3 inflammasome-mediated gout pathogenesis and exerted renoprotective effects in hyperuricemic rat models (127).

In conclusion, sweet tea exhibited considerable potential for the prevention and treatment of chronic metabolic diseases. Conventional tea consumption predominantly relied on a single class of phytochemicals—catechins—which exerted therapeutic effects against metabolic diseases primarily through the enhancement of glucose metabolism and antioxidant pathways (128). However, catechins possessed negligible sweetness (being predominantly bitter and astringent) and contained caffeine (1–4%), which exerted a central nervous system stimulatory effect (129). In contrast, sweet tea contained negligible caffeine and was therefore more suitable for patients with metabolic diseases. Furthermore, sweet tea not only contained the non-sweet bioactive catechins but also encompassed diverse sweet components including dihydrochalcones, diterpene glycosides, and triterpene saponins. The diversity and complexity of these component types not only conferred the bioactivities associated with catechins but also enabled action through more intricate and varied target mechanisms mediated by their sweet components.

## 7 Bibliometric analyses of functional sweet tea

**Figure 9:**
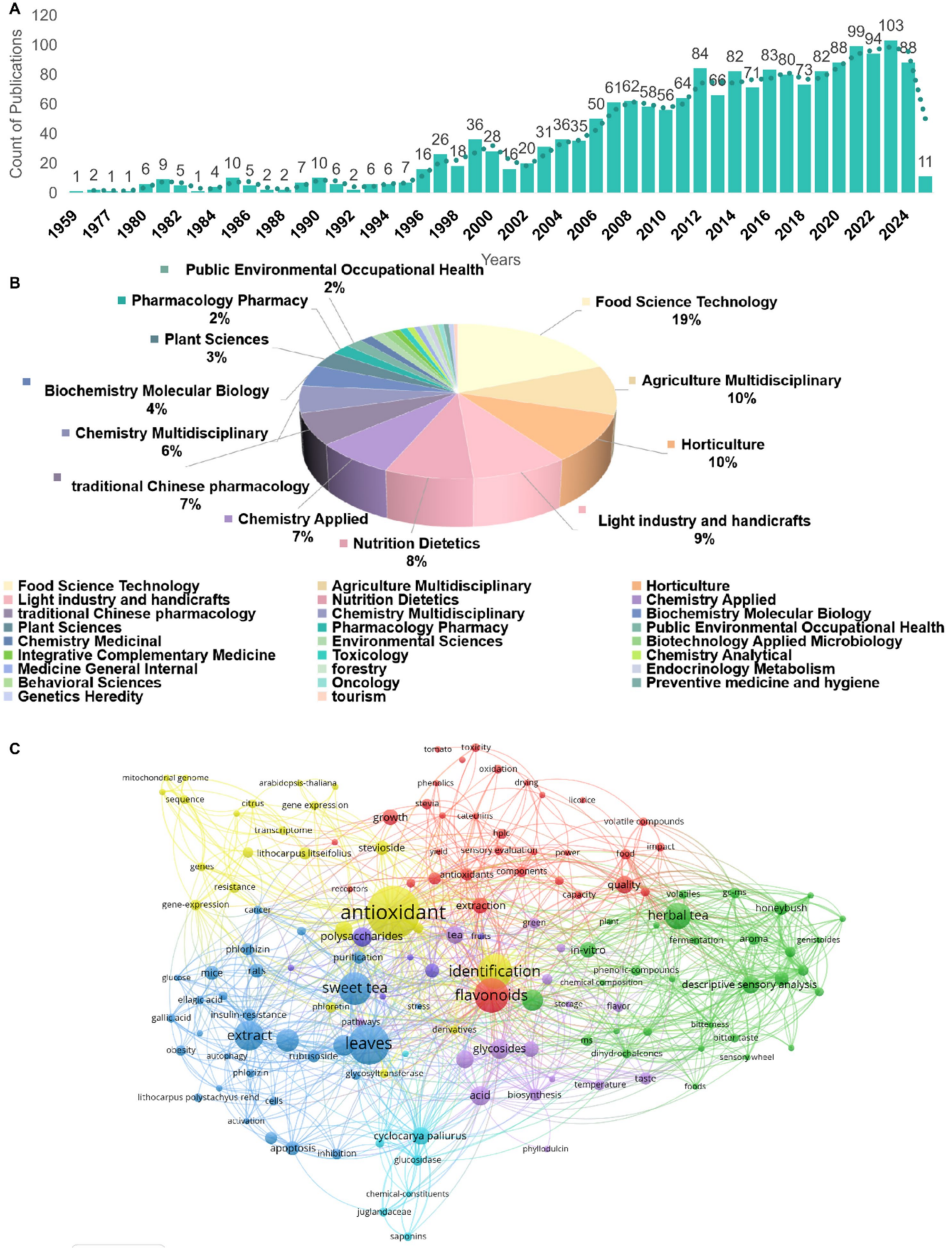
Visual analysis of sweet tea. **(A)** Articles and citations on sweet tea; **(B)** Field of research on sweet tea; **(C)** Keywords cluster analysis.

By using Excel and bibliometric software VOSviewer, we conducted trend analyses and cluster analyses on the number of publications and keywords related to sweet tea (Figure 9A). In 1959, the earliest reports article on sweet tea dated back to a Chinese report published. Analysis of publications from 1959 to 2025 revealed a steady annual increase in sweet tea research. In 2006, the number of published reached 50. In 2023, the number of published articles peaked 103.

Sweet tea research spanned nearly 10 disciplines, including food science and technology, agriculture, horticulture, nutritional balance, phytochemistry, molecular biology, pharmacology, and other scientific and technical fields. Notably, current research on sweet tea predominantly focused on food science and technology, with pharmacological activity research accounting for only 3% (Figure 9B), and pharmacological research primarily focused on the antioxidant, anti-inflammatory, and hypoglycemic properties of *C. paliurus*, *L. litseifolius*, *R. chingii* var. *suavissimus*, *S. grosvenorii*, *G. uralensis* and *S. rebaudiana*. However, significant gaps remained in the pharmacological activity research of other sweet tea species. The cluster diagram (Figure 9C) revealed that all keywords could be categorized into six clusters, with the most frequent terms being sweet tea (leaf/total extract), herbal tea, glycosides, flavonoids, Cyclocarya (*C. paliurus*), and antioxidant. This demonstrated that most sweet tea research primarily focused on *C. paliurus*, *L. litseifolius*, *R. chingii* var. *suavissimus*, and *S. rebaudiana*, particularly their leaves, total extracts, flavonoid components and their antioxidant bioactivities. This demonstrated that most sweet tea research primarily focused on *C. paliurus*, *L. litseifolius*, *R. chingii* var. *suavissimus*, and *S. rebaudiana*, particularly their leaves, total extracts, flavonoid components and their antioxidant bioactivities.

## 8 Opportunities and challenges in the functional sweet tea development

Due to high sweetness, low caloric content, and broad health benefits, sweet tea has garnered increasing attention from scholars worldwide. It holds significant potential for development in pharmaceuticals, confectionery, beverages, and health products, and is anticipated to become an ideal sweetener for patients with chronic metabolic diseases such as hyperglycemia and hyperlipidemia. Nevertheless, the research and development of sweet tea continue to face substantial challenges:

(1) The botanical sources of sweet tea exhibit considerable diversity, and the sweet components of some sweet tea is unclear: while we have systematically presented the sweet components and health benefits of various sweet tea species, the bioactive sweet components in certain species remain uncharacterized. Future research should prioritize accurate phenotypic identification of sweet tea species and whole-genome germplasm characterization, elucidate enzymes catalyzing or regulating sweet component biosynthesis, facilitate efficient biosynthetic production, establish green enrichment processes, and employ mass spectrometry-based strategies to discover novel sweet component. The development of a “variety-component-quality” correlation database would provide a more systematic and robust scientific foundation for the comprehensive development of sweet tea products, thereby enhancing product quality and generating value-added opportunities.
(2) Lack of standardized sweetness evaluation: although we have summarized and predicted the sweetness of known sweet components, most sweet teas are only roughly compared to sucrose in terms of sweetness without other related affecting factors. We should employ multidisciplinary approaches integrating structural similarity analysis with sucrose, such as electronic tongue technology, and artificial intelligence to elucidate sweet taste receptors and flavor omics profiles.
(3) Limited molecular mechanisms of health benefits of sweet tea: Current pharmacological research methodologies remain relatively rudimentary, predominantly relying on confirmatory studies of established pathways—including glycolipid metabolism, anti-inflammatory responses, and oxidative stress mitigation—while lacking depth in mechanistic elucidation and discovery of novel regulatory pathways. Through the integration of metabolomics, transcriptomics, and proteomics analyses (1), the multi-target regulatory networks and mechanisms of action of sweet tea and its sweet component can be systematically elucidated. For instance, metabolomics can be employed to analyze the *in vivo* metabolic fate of sweet tea constituents, network pharmacology can predict the molecular targets of active components, and integrated omics approaches can uncover additional signaling pathways modulated by sweet tea and its constituents. Concurrently, molecular docking and cellular thermal shift assays should be employed to validate target binding and confirm therapeutic targets.
(4) Clinical evidence for the health benefits of sweet tea is deficient: Current research has predominantly focused on *in vitro* studies and animal models, with a marked paucity of clinical trials. Clinical evidence substantiating the efficacy of sweet tea and its sweet component in the prevention and treatment of chronic metabolic diseases remains critically insufficient. Most trials have durations of ≤12 weeks, median sample sizes below 100 participants, and consequently inadequate statistical power. Dose–response relationships remain poorly defined, and few trials have incorporated predefined dose–response cohorts or biomarker-anchored exposure metrics. Data pertaining to special populations—including pregnant women, children, and individuals with metabolic syndrome—are particularly scarce (2). Furthermore, only a limited number of sweet tea species—namely *Stevia rebaudiana* and Glycyrrhiza species—have undergone partial mechanistic validation in humans. Future research should prioritize the development of human sample-based validation models to bridge the translational gap between animal and human studies, alongside intensified investigations into dose conversion, pharmacokinetic profiles, and metabolic pathway differences across species. Specific approaches include: (i) mechanism validation using human-derived tissues (e.g., intestinal organoids); (ii) development of humanized animal models to enhance preclinical predictive accuracy; (iii) design of dose-escalation Phase I clinical trials to establish human safety dose ranges; and (iv) implementation of rigorous randomized controlled trials with systematic data collection and analysis frameworks to accelerate the research, development, and application of sweet tea-based health foods and pharmaceuticals.
(5) Sweet tea-related products predominantly exist as primary agricultural commodities or as tea products from local enterprises, without an established long-term safety monitoring framework. Product development has largely concentrated on crude extracts, with comparatively little effort devoted to the refinement and application of their sweet components. Future development should prioritize the advancement of health supplements and functional foods derived from the natural sweet components of sweet tea. This strategy should aim to consolidate the dominant position of mogrosides and steviol glycosides in the global natural high-intensity sweetener market. Concurrently, efforts should be directed toward diversifying the product formats of “substitute teas” derived from species such as *C. paliurus*, *N. grossedentata*, *N. megalophylla*, *N. cantoniensis* (vine tea), and *L. litseifolius*. Furthermore, sweet components such as glycyrrhizin, phlorizin and dihydromyricetin warrant further development into high-value dietary supplements to address the convenience and precise dosing requirements of specific consumer populations (e.g., individuals with prediabetes, fatty liver disease, or chronic alcohol consumption). For approved natural sweeteners and sweet tea products, a long-term post-marketing safety surveillance system should be established, with particular attention to their chronic effects on cardiovascular function, gut microbiota composition, and metabolic health. Concurrently, a natural sweetener consumption registry should be established to track health outcomes among long-term users, and guidelines with usage limits for special populations should be developed.

In the context of global sugar reduction and functionalization emerging as definitive trends in the food and beverage industry, the systematic synthesis of sweet tea species and sweet component has established a “constituent-function-process” scientific foundation for product development. Concurrently, research on anti-metabolic disease mechanisms has catalyzed the transformation of natural sweeteners from passive sugar substitutes into active health promoters. This research paradigm constitutes a core driving force for the standardization, internationalization, and premiumization of the sweet tea industry.

## 9 Concluding remarks

Diversified sweet tea has the functions of tea, food and medicine with significant commercial potential, which is not only a natural healthy drink, but also an excellent raw material for sugar substitute and innovative medicine of chronic metabolic diseases. We systematically summarized 22 sweet tea species and their 27 sweet components, which could guide the use of sweet tea germplasm and promote the cultivation of standard and efficient sweet tea. These 27 sweet components showed strong potential for the health benefits of chronic metabolic diseases through multiple pathways such as anti-inflammation, antioxidant and improvement of glycolipid metabolism. We hope that this systematic review will provide new insights into the development of sweet tea resources and applications in chronic disease management, and suggest directions for further research and development.

## Data Availability

The original contributions presented in the study are included in the article/supplementary material, further inquiries can be directed to the corresponding author/s.

## Footnotes

1
http://chemsweet.ddai.tech/services/prediction_result

## Glossary

*G. uralensis*: *Glycyrrhiza uralensis Fisch.*
*A. precatorius*: *Abrus precatorius L.*
*H. strigosa*: *Hydrangea strigosa Rehder*
*H. chinensis*: *Hydrangea chinensis Maxim.*
*S. grosvenorii*: *Siraitia grosvenorii (Swingle) C. Jeffrey ex A. M. Lu & Zhi Y. Zhang*
*E. roxburghiana*: *Engelhardia roxburghiana Wall.*
*C. paliurus*: *Cyclocarya paliurus (Batalin) Iljinsk.*
*S. rebaudiana*: *Stevia rebaudiana (Bertoni) Bertoni*
*N. grossedentata*: *Nekemias grossedentata (Hand.-Mazz.) J. Wen & Z. L. Nie*
*N. megalophylla*: *Nekemias megalophylla (Diels & Gilg) J. Wen & Z. L. Nie*
*N. cantoniensis*: *Nekemias cantoniensis (Hook. & Arn.) J. Wen & Z. L. Nie*
*M. sinensis*: *Mycetia sinensis (Hemsl.) Craib*
*H. cantoniensis*: *Hedyotis cantoniensis F. C. How ex W. C. Ko*
*H. mellii*: *Hedyotis mellii Tutcher*
*H. hedyotidea*: *Hedyotis hedyotidea (DC.) Merr.*
*H. acutangula*: *Hedyotis acutangula Champ. ex Benth.*
*L. litseifolius*: *Lithocarpus litseifolius (Hance) Chun*
*M. hupehensis* var. *mengshanensis*: *Malus hupehensis var. mengshanensis G. Z. Qian & W. H. Shao*
*R. chingii* var. *suavissimus*: *Rubus chingii var. suavissimus (S. Lee) L. T. Lu*
*B. flavescens*: *Berchemia flavescens (Wall.) Brongn.*
*D. gracilentum*: *Decaspermum gracilentum (Hance) Merr. & L. M. Perry*
*S. dulcis*: *Scoparia dulcis L.*
U.S. FDA: U.S. Food and Drug Administration
α-glu: α-glucosidase
IR: insulin receptor
IRS: insulin receptor substrate
GSK-3β: glycogen synthase kinase 3β phosphorylation
AMPK: adenosine monophosphate-activated protein kinase
PI3K: phosphatidylinositol 3-kinase
AKT: protein kinase B
GLUT4: glucose transporter 4
G-6-P: glucose-6-phosphatase
SGLT: sodium-dependent glucose transporter
HMGB1: high mobility group box 1
TLR4: toll-like receptor 4
NF-κB: nuclear factor-κB
TNF-α: anti-tumor necrosis factor-α
IL: interleukin
NLRP3: NOD-like receptor family pyrin domain containing 3
ROS: reactive oxygen species
SOD: antioxidant enzymes
Nrf2: nuclear factor erythroid 2-related factor 2
HO-1: oxygenase-1
FXR: farnesoid X receptor
AST: aspartate aminotransferase
ALT: alanine aminotransferase
SNMC: stronger neo-minophagen C
PGC-1α: proliferator-activated receptor gamma coactivator 1α
PPAR: peroxisome proliferators-activated receptor
XOD: xanthine oxidase
